# Immune profiling of SARS-CoV-2 infection during pregnancy reveals NK cell and **γδ** T cell perturbations

**DOI:** 10.1172/jci.insight.167157

**Published:** 2023-04-10

**Authors:** Jennifer R. Habel, Brendon Y. Chua, Lukasz Kedzierski, Kevin J. Selva, Timon Damelang, Ebene R. Haycroft, Thi H.O. Nguyen, Hui-Fern Koay, Suellen Nicholson, Hayley A. McQuilten, Xiaoxiao Jia, Lilith F. Allen, Luca Hensen, Wuji Zhang, Carolien E. van de Sandt, Jessica A. Neil, Katherine Pragastis, Jillian S.Y. Lau, Jaycee Jumarang, E. Kaitlynn Allen, Fatima Amanant, Florian Krammer, Kathleen M. Wragg, Jennifer A. Juno, Adam K. Wheatley, Hyon-Xhi Tan, Gabrielle Pell, Susan Walker, Jennifer Audsley, Arnold Reynaldi, Irani Thevarajan, Justin T. Denholm, Kanta Subbarao, Miles P. Davenport, P. Mark Hogarth, Dale I. Godfrey, Allen C. Cheng, Steven Y.C. Tong, Katherine Bond, Deborah A. Williamson, James H. McMahon, Paul G. Thomas, Pia S. Pannaraj, Fiona James, Natasha E. Holmes, Olivia C. Smibert, Jason A. Trubiano, Claire L. Gordon, Amy W. Chung, Clare L. Whitehead, Stephen J. Kent, Martha Lappas, Louise C. Rowntree, Katherine Kedzierska

**Affiliations:** 1 Department of Microbiology and Immunology, University of Melbourne, Peter Doherty Institute for Infection and Immunity, Melbourne, Victoria, Australia.; 2 Global Institution for Collaborative Research and Education (GI-CoRE), Hokkaido University, Sapporo, Japan.; 3 Faculty of Veterinary and Agricultural Sciences, University of Melbourne, Melbourne, Victoria, Australia.; 4 Victorian Infectious Diseases Reference Laboratory, Royal Melbourne Hospital, Peter Doherty Institute for Infection and Immunity, Melbourne, Victoria, Australia.; 5 Department of Infectious Diseases, Alfred Health, Monash University, Melbourne, Victoria, Australia.; 6 Department of Infectious Diseases, Eastern Health, Box Hill, Victoria, Australia.; 7 Division of Infectious Diseases, Children’s Hospital Los Angeles, Los Angeles, California, USA.; 8 Department of Immunology, St. Jude Children’s Research Hospital, Memphis, Tennessee, USA.; 9 Department of Microbiology, and; 10 Graduate School of Biomedical Sciences, Icahn School of Medicine at Mount Sinai, New York, New York, USA.; 11 ARC Centre of Excellence in Convergent Bio-Nano Science and Technology, University of Melbourne, Melbourne, Victoria, Australia.; 12 Mercy Perinatal Research Centre, Mercy Hospital for Women, Heidelberg, Victoria, Australia.; 13 Department of Infectious Diseases, University of Melbourne, Peter Doherty Institute for Infection and Immunity, Melbourne, Victoria, Australia.; 14 Kirby Institute, University of New South Wales, Sydney, New South Wales, Australia.; 15 Victorian Infectious Diseases Service, Royal Melbourne Hospital, Peter Doherty Institute for Infection and Immunity, Melbourne, Victoria, Australia.; 16 WHO Collaborating Centre for Reference and Research on Influenza, Peter Doherty Institute for Infection and Immunity, Melbourne, Victoria, Australia.; 17 Immune Therapies Laboratory, Burnet Institute, Melbourne, Victoria, Australia.; 18 Department of Immunology and Pathology, Central Clinical School, Monash University, Melbourne, Victoria, Australia.; 19 Department of Clinical Pathology, University of Melbourne, Parkville, Victoria, Australia.; 20 School of Public Health and Preventive Medicine, Monash University, Melbourne, Victoria, Australia.; 21 Infection Prevention and Healthcare Epidemiology Unit, Alfred Health, and Monash Infectious Diseases, Monash Health, Melbourne, Victoria, Australia.; 22 Department of Infectious Diseases, The University of Melbourne at the Peter Doherty Institute for Infection and Immunity, Melbourne, Australia.; 23 Department of Microbiology, Royal Melbourne Hospital, Peter Doherty Institute for Infection and Immunity, Melbourne, Victoria, Australia.; 24 Departments of Pediatrics, Molecular Microbiology and Immunology, Keck School of Medicine, UCLA, Los Angeles, California, USA.; 25 Department of Infectious Diseases, Austin Health, Heidelberg, Victoria, Australia.; 26 Department of Critical Care, University of Melbourne, Parkville, Victoria, Australia.; 27 Data Analytics Research and Evaluation Centre, Austin Health, University of Melbourne, Heidelberg, Victoria, Australia.; 28 Centre for Antibiotic Allergy and Research, Department of Infectious Diseases, Austin Health, Heidelberg, Victoria, Australia.; 29 Department of Infectious Diseases, and; 30 National Centre for Infections in Cancer, Peter McCallum Cancer Centre, Melbourne, Victoria, Australia.; 31 Department of Medicine (Austin Health), University of Melbourne, Heidelberg, Victoria, Australia.; 32 Department of Obstetrics and Gynaecology, University of Melbourne, Parkville, Victoria, Australia.; 33 Pregnancy Research Centre, Royal Women’s Hospital, Parkville, Victoria, Australia.; 34 Melbourne Sexual Health Centre, Infectious Diseases Department, Alfred Health, Central Clinical School, Monash University, Melbourne, Victoria, Australia.; 35 Obstetrics, Nutrition and Endocrinology Group, Department of Obstetrics and Gynaecology, University of Melbourne, Victoria, Australia.

**Keywords:** Immunology, Infectious disease, Innate immunity, NK cells, T cells

## Abstract

Pregnancy poses a greater risk for severe COVID-19; however, underlying immunological changes associated with SARS-CoV-2 during pregnancy are poorly understood. We defined immune responses to SARS-CoV-2 in unvaccinated pregnant and nonpregnant women with acute and convalescent COVID-19, quantifying 217 immunological parameters. Humoral responses to SARS-CoV-2 were similar in pregnant and nonpregnant women, although our systems serology approach revealed distinct antibody and FcγR profiles between pregnant and nonpregnant women. Cellular analyses demonstrated marked differences in NK cell and unconventional T cell activation dynamics in pregnant women. Healthy pregnant women displayed preactivated NK cells and γδ T cells when compared with healthy nonpregnant women, which remained unchanged during acute and convalescent COVID-19. Conversely, nonpregnant women had prototypical activation of NK and γδ T cells. Activation of CD4^+^ and CD8^+^ T cells and T follicular helper cells was similar in SARS-CoV-2–infected pregnant and nonpregnant women, while antibody-secreting B cells were increased in pregnant women during acute COVID-19. Elevated levels of IL-8, IL-10, and IL-18 were found in pregnant women in their healthy state, and these cytokine levels remained elevated during acute and convalescent COVID-19. Collectively, we demonstrate perturbations in NK cell and γδ T cell activation in unvaccinated pregnant women with COVID-19, which may impact disease progression and severity during pregnancy.

## Graphical abstract

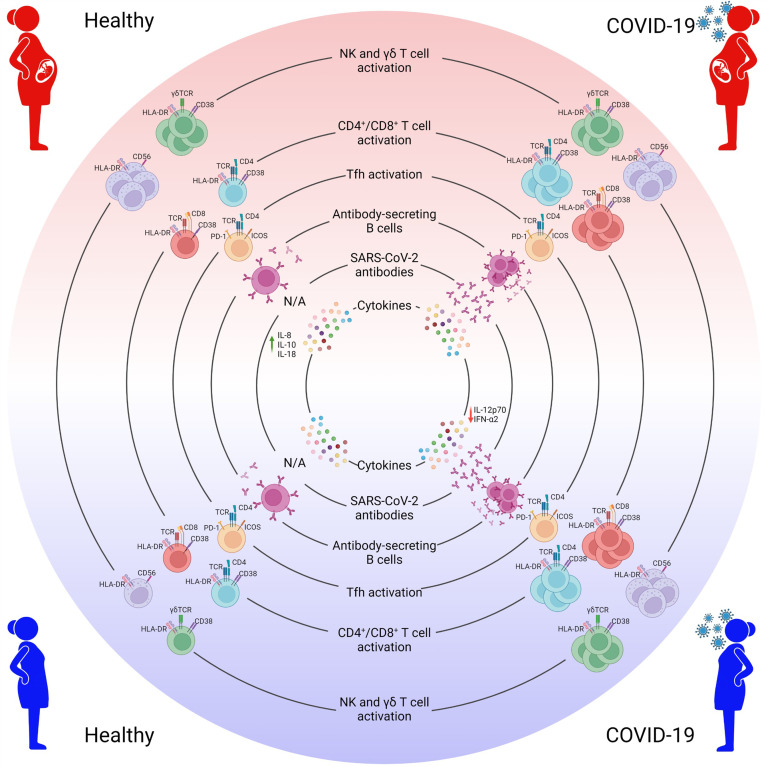

## Introduction

SARS-CoV-2 emerged in late 2019, causing a pandemic that has resulted in hundreds of millions of infections and more than 6 million deaths globally (1). Understanding immune responses to SARS-CoV-2, especially in high-risk groups, is of critical importance to guide treatment and vaccine strategies. The majority of immunological studies on COVID-19 largely focused on correlates of disease severity in previously healthy individuals across different age groups (2–4). Most COVID-19 patients develop prototypical broad, robust, and transient antiviral immune responses (2, 3, 5–8), with abundant SARS-CoV-2–specific antibodies, B cell, and T cell responses (4, 9–12), and establishment of long-lasting immune memory (4, 13–17). Hyperactivation of innate/adaptive immune responses and blood hypercytokinemia are characteristic of severe disease (2, 5, 8, 18). There are, however, limited data on immunity to SARS-CoV-2 infection in groups vulnerable to poor health outcomes following infection in the absence of vaccination, especially pregnant women.

Pregnant women are considered a vulnerable group for SARS-CoV-2 infection due to physiological and immunological changes occurring during gestation (19). Studies to date associate COVID-19 during pregnancy with increased risk of intensive care unit (ICU) admission, invasive ventilation, and extracorporeal membrane oxygenation (ECMO), compared with nonpregnant women of reproductive age (19, 20). Pregnant women with COVID-19 are at increased risk of death, sepsis, mechanical ventilation, ICU admission, shock, acute renal failure, and thromboembolic disease (21). Additionally, COVID-19 during pregnancy has been linked to increased risk of preeclampsia and gestational hypertension (22), maternal morbidity, preterm birth, and venous thromboembolism compared with healthy pregnancies (23). Nonetheless, others showed that pregnant women commonly have mild or asymptomatic SARS-CoV-2 infection (24); however, gestational immune alterations may impair antiviral responses, leading to severe disease (25–29).

Published evidence shows that pregnant women with COVID-19 produced SARS-CoV-2–specific antibodies, and that SARS-CoV-2–specific IgG is transferred to cord blood (30–32). RNA sequencing in COVID-19 convalescent pregnant and nonpregnant women revealed key differences in NK, NKT, and mucosal-associated invariant T (MAIT) cells, suggesting increased activation during pregnancy (33). Additionally, higher levels of low-density neutrophils were found in pregnant women with mild or asymptomatic SARS-CoV-2 infection, a characteristic typically observed in patients with severe COVID-19 (34). However, a comprehensive analysis of antibody, cytokine, and immune cell activation phenotypes in early and late stages of COVID-19 during pregnancy, in comparison with healthy pregnancies and nonpregnant women, is lacking.

Our present study fills this knowledge gap by investigating in-depth innate, adaptive, and humoral immune responses to SARS-CoV-2 in pregnant women. We examined samples from 119 women to define SARS-CoV-2–specific immunity in pregnant and nonpregnant women during acute and convalescent COVID-19, encompassing days 1–258 after disease onset and quantifying 217 immunological parameters. This provides the first comprehensive map to our knowledge of immune responses in pregnant women during acute and convalescent phases of SARS-CoV-2 infection. Our longitudinal comparisons revealed a lack of γδ T cell, MAIT, and NK cell activation in pregnant women during acute COVID-19, as a result of their preactivated profile during the healthy state. Conversely, activation of classical αβ CD4^+^ and CD8^+^ T cells, T follicular helper (Tfh), antibody-secreting cells (ASCs), and SARS-CoV-2–specific antibody patterns were similar between pregnant and nonpregnant women. Differences in IL-8, IL-10, and IL-18 levels were evident during healthy pregnancy, and these cytokines remained elevated during acute and convalescent COVID-19. Overall, our comprehensive analysis of immune dysfunction following COVID-19 in pregnancy provides key insights into immune responses during pregnancy, which can potentially inform patient management and education.

## Results

### COVID-19 pregnancy cohort demographics.

**Figure 1:**
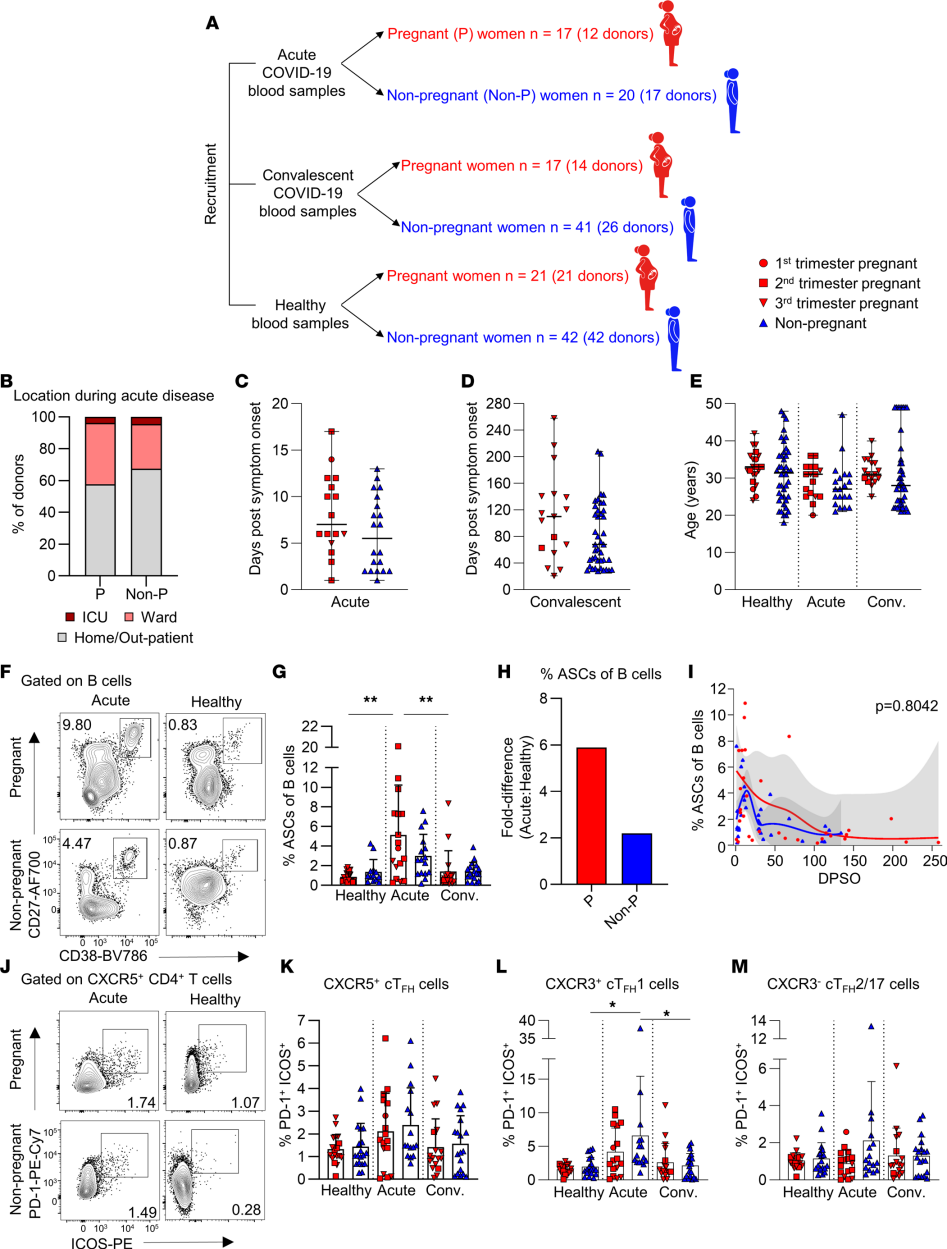
COVID-19 pregnancy cohort and activation in antibody-secreting B cells and Tfh cells. (**A**) Schematic depicting blood sample collection of pregnant and nonpregnant women with acute or convalescent COVID-19 or healthy individuals not exposed to SARS-CoV-2. (**B**) Proportions of pregnant (P) and nonpregnant (Non-P) women at different locations during acute COVID-19. (**C** and **D**) Median DPSO in (**C**) acute (P, *n* = 16; Non-P, *n* = 20) and (**D**) convalescent (P, *n* = 17; Non-P, *n* = 41) pregnant and nonpregnant donors. Donors with longitudinal sampling are represented for each time point collected. (**E**) Median age of pregnant and nonpregnant healthy (*n* = 21 and 42), acute (*n* = 17 and 20), or convalescent (*n* = 17 and 41) COVID-19 donors. Donors with longitudinal sampling are represented for each time point collected. (**F**) Antibody-secreting cells (ASCs) were defined as CD27^+^CD38^+^ from the CD19^+^CD3^–^ B cell population. AF700, Alexa Fluor 700. (**G**) Frequencies of ASCs of B cells in healthy (P, *n* = 18; Non-P, *n* = 13), acute (P, *n* = 17; Non-P, *n* = 16), or convalescent (P, *n* = 17; Non-P, *n* = 18) pregnant or nonpregnant women. (**H**) Fold difference in the mean frequency of ASCs between healthy and acute COVID-19 for pregnant and nonpregnant donors. (**I**) LOESS regression kinetics of ASCs in pregnant and nonpregnant women with COVID-19. The 95% CI is shown in gray. (**J**) Tfh cells were defined as CXCR5^+^CD4^+^ T cells and activation determined by PD-1 and ICOS expression. (**K**–**M**) Frequencies of PD-1^+^ICOS^+^ of (**K**) total Tfh cells, (**L**) CXCR3^+^ Tfh1 cells, and (**M**) CXCR3^–^ Tfh2/17 cells in healthy (P, *n* = 18; Non-P, *n* = 19), acute (P, *n* = 17; Non-P, *n* = 16), or convalescent (P, *n* = 17; Non-P, *n* = 19) pregnant or nonpregnant women. Median and range are shown in **C**–**E**. Means and SDs are shown in **G** and **K**–**M**. Significance determined by Mann-Whitney *U* test (**C** and **D**), Kruskal-Wallis test (**E**, **G**, and **K**–**M**), or Wald’s test (**I**). *P < 0.05; **P < 0.01.

We analyzed 119 women to define cellular and humoral immune responses to SARS-CoV-2 during pregnancy (Supplemental Tables 1 and 2; supplemental material available online with this article; https://doi.org/10.1172/jci.insight.167157DS1). Acute and convalescent COVID-19 blood samples were collected from 23 pregnant women with PCR-confirmed SARS-CoV-2 infection during pregnancy (acute *n* = 12; convalescent *n* = 14) and 33 nonpregnant women (acute *n* = 17; convalescent *n* = 26) (Figure 1A). Healthy control blood samples were collected from 21 pregnant and 42 nonpregnant women, with no history of SARS-CoV-2 infection. All SARS-CoV-2 infections occurred while the ancestral or Alpha variants were in circulation. Similar proportions of pregnant and nonpregnant women were located at home (57.7% vs. 67.4%, respectively), in hospital ward (38.4% vs 27.9%), or in the ICU (3.8% vs 4.7%) (Figure 1B). Blood from pregnant and nonpregnant women with acute COVID-19 was collected from 1 to 17 and 1 to 13 days post symptom onset (DPSO), respectively (Figure 1C). One asymptomatic pregnant woman had a sample collected 1 day after her SARS-CoV-2 PCR-positive result. Convalescent blood samples from pregnant and nonpregnant women were collected from 21 to 258 and 28 to 208 DPSO, respectively (Figure 1D). There were no significant differences in timing of sample collection between acute pregnant and nonpregnant groups (median of 7 and 5.5 days, respectively) and convalescent pregnant and nonpregnant groups (median of 110 and 68 days). The ages of pregnant and nonpregnant women in the healthy, acute, or convalescent groups were similar, ranging from 18 to 49 years (Figure 1E).

### Similar frequencies of antibody-secreting B cells and activated circulating Tfh cells in pregnant and nonpregnant COVID-19.

ASCs were analyzed to determine B cell activation in COVID-19 pregnant and nonpregnant women (Figure 1F). Comparable frequencies of ASCs were observed in pregnant and nonpregnant women with acute (mean 5.1% and 3.0%, respectively) and convalescent (mean 1.4% and 1.4%) COVID-19 (Figure 1G). While frequencies between pregnant and nonpregnant women were similar, the mean fold difference in ASC frequency between healthy and acute disease groups was approximately 6-fold higher in pregnant compared with an approximately 2-fold difference in nonpregnant women (Figure 1H), although ASCs displayed similar kinetics in both groups (Figure 1I).

Circulating CXCR5^+^ Tfh (cTfh) CD4^+^ T cells correlate with B cell maturation and activation (35). Total cTfh cell activation (PD-1^+^ICOS^+^) was similar between COVID-19 pregnant and nonpregnant women (Figure 1, J and K). Further subsetting of cTfh into CXCR3^+^ type 1 (cTfh1) and CXCR3^–^ type 2/17 (cTfh2/17) revealed a dynamic change in the frequency of activated cTfh1 cells in nonpregnant women, in whom a significant increase occurred during acute infection followed by a decrease at convalescence (Figure 1L). Similar activation levels of the cTfh2/17 subset were observed in pregnant and nonpregnant women across all disease states (Figure 1M).

### Comparable levels of RBD-specific, N-specific, and neutralizing antibodies in pregnant and nonpregnant women.

**Figure 2:**
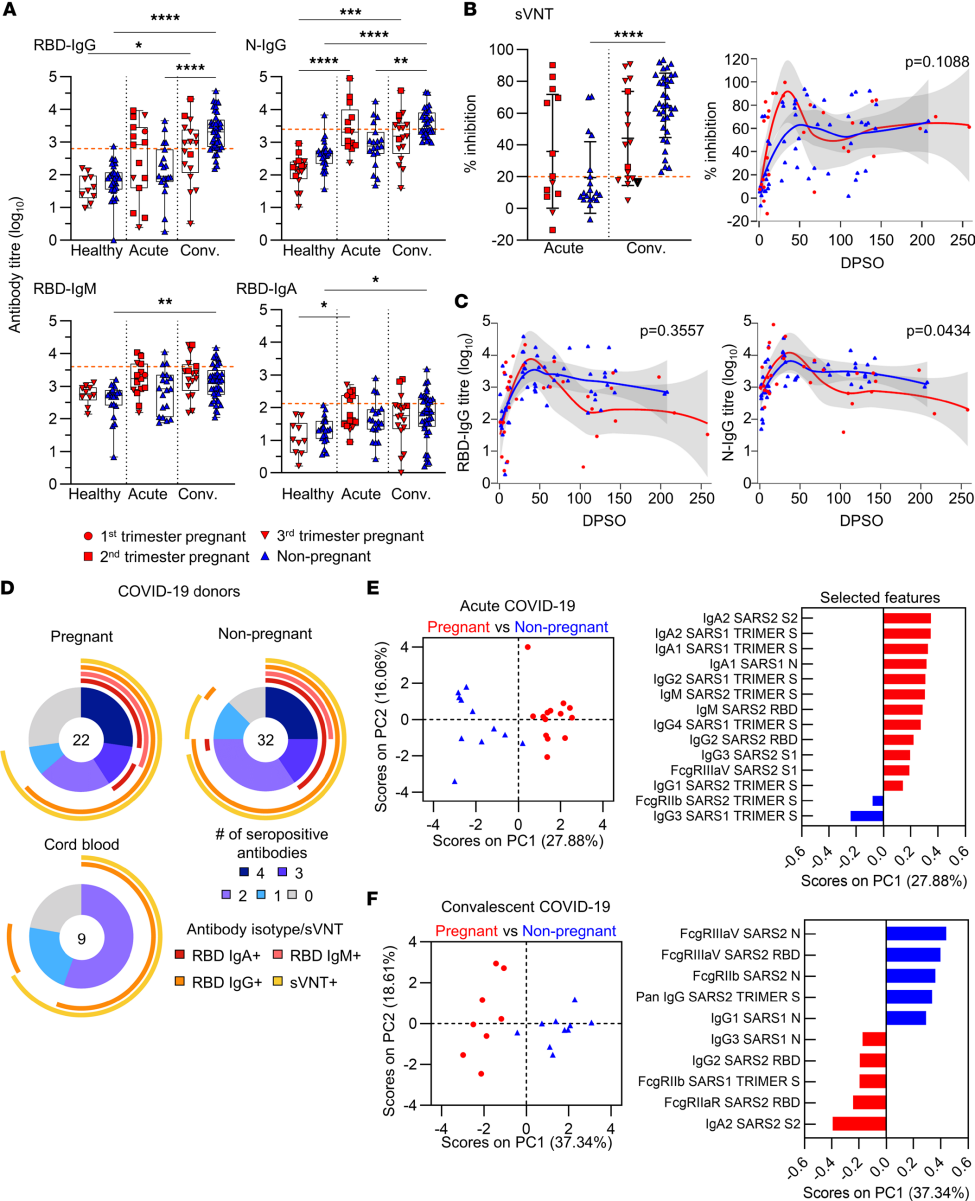
Analysis of SARS-CoV-2–specific antibodies in pregnant and nonpregnant women. (**A**) Log_10_-transformed RBD-specific or N-specific antibody titers in pregnant (P) and nonpregnant (Non-P) healthy (P, *n* = 10–16; Non-P, *n* = 21–31), acute COVID-19 (P, *n* = 13–15; Non-P, *n* = 19), and convalescent COVID-19 (P, *n* = 17; Non-P, *n* = 31–41) donors. Orange dashed lines indicate seroconversion cutoff calculated from the mean + 2 SD of the healthy pregnant and nonpregnant titers. (**B**) sVNT percentage inhibition in acute (P, *n* = 13; Non-P, *n* = 11) and convalescent (P, *n* = 15; Non-P, *n* = 28) pregnant or nonpregnant women. Seropositivity indicated by orange dashed line. (**C**) LOESS regression of RBD- and N-IgG kinetics for pregnant (red) and nonpregnant (blue) COVID-19 donors. The 95% CI is shown in gray in **B** and **C**. (**D**) Proportions of pregnant, nonpregnant, and cord blood donors who seroconverted with different combinations of RBD-specific or neutralizing antibodies. Seroconversion counted if a donor had a positive readout at any time point if longitudinal samples were collected. (**E**) Principal component plots showing pregnant (red, *n* = 13) and nonpregnant (blue, *n* = 11) donors with acute COVID-19 (left). Loading plot showing features separating pregnant and nonpregnant donors along the PC1 axis (right). (**F**) Principal component plots showing pregnant (red, *n* = 8) and nonpregnant (blue, *n* = 10) donors with convalescent COVID-19 (left). Loading plot showing features separating pregnant and nonpregnant donors along the PC1 axis (right). SARS2, SARS-CoV-2; SARS1, SARS-CoV-1; S, spike; S1, spike subunit 1 (head); S2, spike subunit 2 (stalk); N, nucleocapsid; RBD, receptor binding domain. Means and SDs are shown in **A** and **B**. Significance determined with a Kruskal-Wallis test (**A** and **B** [left]) or with Wald’s test (**B** [right] and **C**) *P < 0.05; **P < 0.01; ***P < 0.001; ****P < 0.0001.

As SARS-CoV-2 antibodies are associated with protection from repeated infection (36), we assessed humoral responses through detection of receptor binding domain–specific (RBD-specific) IgG, IgM, and IgA, and nucleocapsid-specific (N-specific) IgG antibodies by ELISA (2, 11) (Figure 2A and Supplemental Figure 1A), while a surrogate virus neutralization test (sVNT) and microneutralization assay measured neutralizing activity of SARS-CoV-2–specific antibodies (16, 37, 38) (Figure 2B and Supplemental Figure 1B). Overall, similar RBD-specific IgG, IgM, or IgA, N-specific IgG titers (Figure 2A), and neutralizing activity (Figure 2B and Supplemental Figure 1B) between pregnant and nonpregnant women with acute or convalescent COVID-19 were observed. Similarly, the avidity of RBD-specific IgG and IgM was comparable between pregnant and nonpregnant women when a urea-mediated dissociation ELISA was performed for sequential time points (Supplemental Figure 1C). RBD- and N-IgG titers showed comparable kinetics in COVID-19 pregnant and nonpregnant women (Figure 2C). Similar proportions of pregnant and nonpregnant donors were positive for the combined detection of RBD-specific antibodies and/or inhibition of the interaction between the RBD and SARS-CoV-2 receptor, angiotensin-converting enzyme 2 (ACE2) (72.7% and 87.5%, respectively; Figure 2D). The proportion of pregnant and nonpregnant women seroconverting for RBD-IgG increased at convalescence (Supplemental Figure 1D). The similarity in antibody titers between pregnant and nonpregnant women demonstrates that production of SARS-CoV-2–specific antibodies is not impaired during pregnancy, and importantly suggests women who had COVID-19 during pregnancy generate a level of humoral protection.

### Neutralizing antibodies and RBD- and N-specific IgGs cross the placenta into cord blood.

Cord blood plasma from COVID-19 convalescent and SARS-CoV-2–unexposed pregnancies were assessed for neutralizing antibodies, RBD-specific IgG, IgM, and IgA, and N-specific IgG (Figure 2D and Supplemental Figure 1, E–G). COVID-19 cord blood had significantly higher RBD- and N-specific IgG titers (Supplemental Figure 1E). sVNT demonstrated that COVID-19 cord blood contained antibodies with neutralizing activity (mean 38.2% inhibition; Supplemental Figure 1F). The majority of cord blood samples contained RBD-IgG and/or neutralizing antibodies (77.8%) (Figure 2D).

Analysis of matched maternal cord dyads demonstrated RBD- and N-specific IgG, and neutralizing antibody levels correlated between maternal and cord blood. Interestingly, RBD- and N-specific IgG titers were higher in cord blood plasma than matched maternal plasma (Supplemental Figure 1F). RBD-specific IgM and IgA were also measured in the cord blood; however, as these isotypes do not vertically transfer, titers were significantly lower in cord blood compared with maternal blood (Supplemental Figure 1G), and not significantly different from unexposed pregnancy cord blood (Supplemental Figure 1E). Our findings verify reports that SARS-CoV-2 IgG antibodies (30, 32, 39) and neutralizing antibodies (40, 41) cross the placenta, providing a layer of immunity against SARS-CoV-2 infection to the neonate.

Antibody glycosylation patterns can impact antibody effector functions, such as IgG binding to Fcγ receptor IIIa (FcγRIIIa) (42), and a skewing toward galactosylation and sialylation is known to occur during pregnancy (43, 44). To define potential differences in antibody functionality, we assessed glycosylation patterns on total IgG, based on the number of galactose (G), sialic acid (S), and fucose (F) residues. Healthy pregnant women had significantly increased G2S1F and G2F, while G0F, G1, and G1F were significantly reduced compared with healthy nonpregnant women (Supplemental Figure 2, A and B). Only G2S1F and G1F maintained their differences between pregnant and nonpregnant women during acute COVID-19.

Overall, analysis of antibody responses clearly demonstrated generation and persistence of neutralizing antibodies and RBD- and N-specific antibodies in pregnant women, and provided evidence for RBD- and N-specific IgG antibodies detected in cord blood of convalescent mothers.

### Systems serology reveals distinct antibody and FcγR profiles between pregnant and nonpregnant women.

To further characterize SARS-CoV-2–specific antibody responses in pregnant and nonpregnant women, a 102-parameter multiplex bead array was performed (45). A range of SARS-CoV-1 and SARS-CoV-2 spike and N antigens led to the detection of epitope-specific antibody subclasses and isotypes (IgG1-4, IgA1-2, and IgM) and FcγR binding (FcγRIIaH, FcγRIIaR, FcγRIIb, FcγRIIIaV, FcγRIIIaF) (Supplemental Figure 2C and Supplemental Table 3).

Comparing serology of pregnant and nonpregnant women with acute COVID-19 revealed a clear separation between groups on principal component 1 (PC1) (27.88%) (Figure 2E). We found that increases in SARS-CoV-2 spike head–specific IgA2; SARS-CoV-1 trimeric spike–specific IgA1, IgA2, IgG2, and IgG4; SARS-CoV-1 N–specific IgA1; SARS-CoV-2 trimeric spike–specific IgG1 and IgM, RBD-specific IgM and IgG2, and spike stalk–specific IgG3 and FcγRIIIaV-binding were biased toward pregnancy, whereas SARS-CoV-2 trimeric spike–specific FcγRIIb and SARS-CoV-1 trimeric spike–specific IgG3 were increased in nonpregnant women (Figure 2E and Supplemental Table 4). The increases in virus-specific IgAs, IgG2, and IgG4 in pregnant women may therefore indicate passive blocking of antibodies that bind FcγRIII.

To understand whether serological features changed over time, we analyzed convalescent pregnant and nonpregnant donors (Figure 2F). Ten features drove the difference between pregnant and nonpregnant women at convalescence and provided 37.34% variance of groups on PC1 (Figure 2F). Among the features elevated in convalescent nonpregnant women, SARS-CoV-2 N–specific FcγRIIIaV- and FcγRIIb-binding, RBD–specific FcγRIIIaV-binding, spike trimer–specific total IgG, and SARS-CoV-1 N–specific IgG1 dominated (Figure 2F). Conversely, pregnant women displayed more spike stalk–specific IgA2, SARS-CoV-2 RBD–specific IgG2 and FcγRIIaR-binding, spike trimer–specific FcγRIIb-binding, and SARS-CoV-1 N–specific IgG3. IgG2 deficiency was associated with disease severity in pregnant women during pandemic H1N1 influenza virus infection (46). Therefore, the significantly higher spike head–specific IgG2 in convalescent pregnant women (Supplemental Figure 1H) could confer protection for individuals with increased IgG2 levels. Overall, our systems serology approach revealed distinct antibody and FcγR-binding profiles between pregnant and nonpregnant women.

### Differential NK cell activation and polyfunctionality in pregnant women during acute COVID-19.

**Figure 3:**
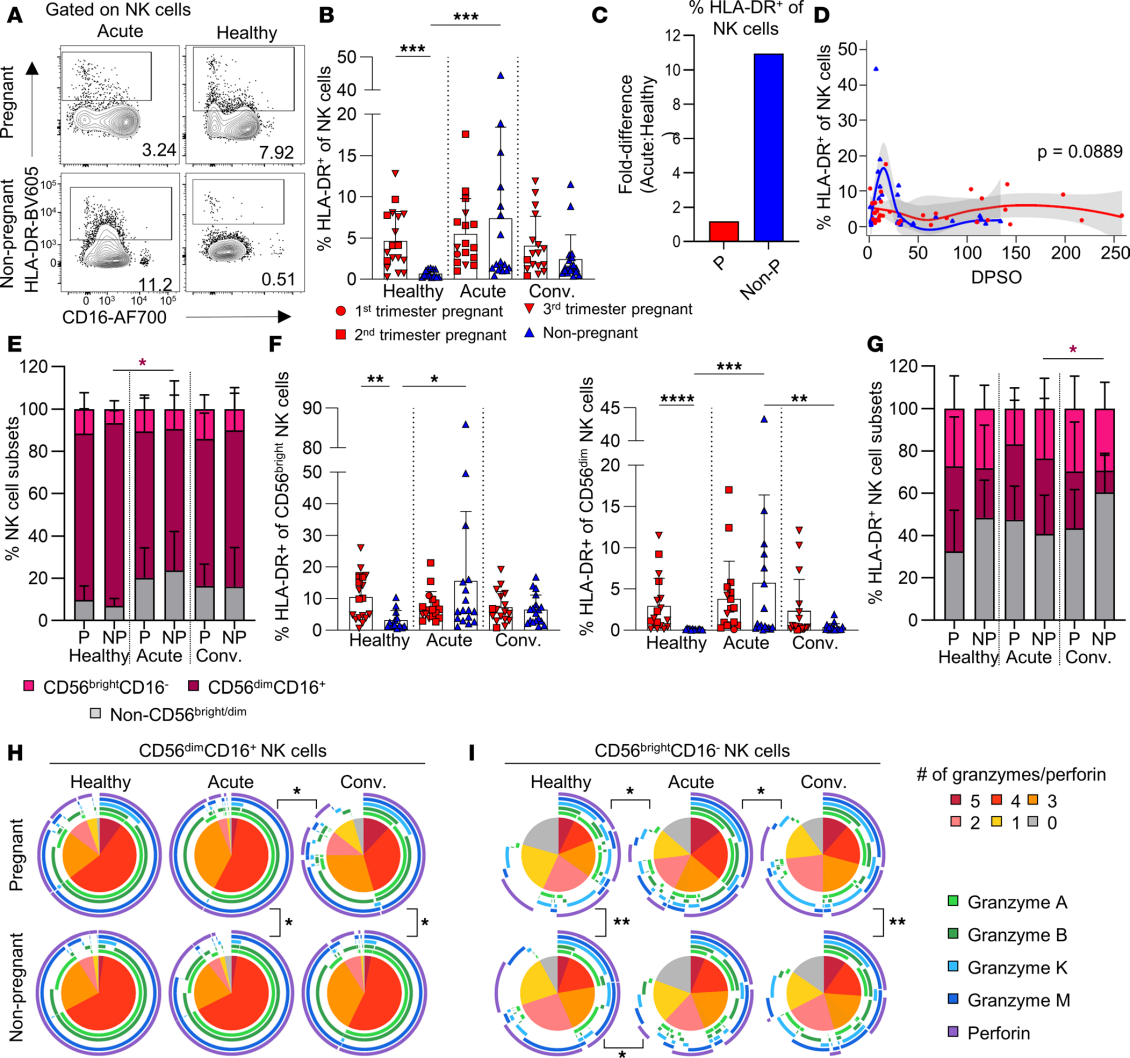
Differential NK cell activation dynamics in pregnant women with COVID-19. (**A**) CD3^–^CD56^+^ NK cell activation by HLA-DR expression. (**B**) HLA-DR^+^ NK cell frequencies in healthy (P, *n* = 18; Non-P, *n* = 13), acute (P, *n* = 17; Non-P, *n* = 17), and convalescent (P, *n* = 17; Non-P, *n* = 18) COVID-19 pregnant and nonpregnant women. (**C**) Fold difference in the mean frequency of HLA-DR^+^ NK cells between healthy and acute groups. (**D**) LOESS regression of HLA-DR^+^ NK cell frequencies and DPSO for pregnant and nonpregnant women with COVID-19. The 95% CI is shown in gray. (**E**) Proportions of CD56^bright^CD16^–^, CD56^dim^CD16^+^, and intermediate non–CD56^bright/dim^ NK cells. (**F**) Frequencies of HLA-DR^+^ CD56^bright^CD16^–^ (left) and CD56^dim^CD16^+^ (right) NK cells in healthy (P, *n* = 18; Non-P, *n* = 13), acute (P, *n* = 17; Non-P, *n* = 17), and convalescent (P, *n* = 17; Non-P, *n* = 18) pregnant and nonpregnant women. (**G**) Proportions of CD56^bright^CD16^–^, CD56^dim^CD16^+^, and intermediate non–CD56^bright/dim^ in HLA-DR^+^ NK cells. (**H** and **I**) Proportions of granzyme and perforin expression in (**H**) CD56^dim^CD16^+^ or (**I**) CD56^bright^CD16^–^ NK cells. Means and SDs are shown in **B** and **E**–**G**. Significance determined by Kruskal-Wallis test (**B** and **E**–**G**), Wald’s test (**D**), or permutations test (**H** and **I**). *P < 0.05; **P < 0.01; ***P < 0.001; ****P < 0.0001.

NK cells play an important role in antiviral immunity, especially mediating rapid killing of virally infected cells. In healthy individuals, pregnant women had significantly higher total NK cell activation based on HLA-DR expression than nonpregnant women (mean 4.7% and 0.7%, respectively, *P* < 0.001) consistent with previous work (47), indicating preactivated NK cells during healthy pregnancy (Figure 3A). Strikingly, activation profiles of these preactivated NK cells remained unchanged in acute and convalescent COVID-19 during pregnancy (Figure 3B). In contrast to nonpregnant women, in whom NK cell activation during acute COVID-19 increased by approximately 11-fold (compared with healthy nonpregnant women), NK activation in pregnant women with acute COVID-19 remained at the level of healthy pregnant participants (fold difference = 1.2; Figure 3C). Differential NK cell activation dynamics between pregnant and nonpregnant women can be visualized by kinetics across longitudinal samples (Figure 3D). Overall, our data reveal a preactivated state of NK cells in healthy pregnancy and tightly regulated processes of NK activation above this preactivated level, resulting in lack of further NK cell activation during acute COVID-19 during pregnancy.

To further define differential NK cell activation between pregnant and nonpregnant women, we divided the NK cell population into CD56^bright^CD16^lo/–^ and CD56^dim^CD16^+^ subsets. CD56^bright^ NK cells are functionally associated with cytokine production, whereas CD56^dim^ NK cells are cytotoxic (48, 49). While there were no differences between pregnant and nonpregnant women regardless of disease status, nonpregnant women with acute COVID-19 had significantly lower CD56^dim^ NK cells than their healthy counterparts (Figure 3E). In parallel with our earlier findings, healthy pregnant women had significantly higher frequencies of HLA-DR^+^CD56^bright^ and HLA-DR^+^CD56^dim^ NK cells compared with healthy nonpregnant women (Figure 3F). CD56^bright^ and CD56^dim^ NK cells comprised similar portions of the HLA-DR^+^ NK cells during a healthy state, whereas there was a significantly higher proportion of CD56^dim^ NK cells compared with CD56^bright^ NK cells during acute disease in pregnant women (Figure 3G and Supplemental Figure 3A).

To assess the functionality of CD56^bright^CD16^lo/–^ and CD56^dim^CD16^+^ NK cell subsets, we performed intracellular staining for granzyme A, B, K, and M, and perforin. CD56^dim^ NK cells largely expressed multiple granzymes/perforin regardless of disease status, consistent with their previously defined cytotoxic function (Figure 3H). Conversely, CD56^bright^ NK cells displayed less multifunctionality overall, but interestingly, CD56^bright^ NK cells displayed less cytotoxicity in healthy pregnant women, while pregnant women with acute COVID-19 had the largest proportions of cells expressing 3–5 cytotoxic molecules, which might indicate increased cytotoxic potential of this classically noncytotoxic NK cell subset during pregnancy (Figure 3I).

### Preactivated γδ T cell phenotype observed during pregnancy.

**Figure 4:**
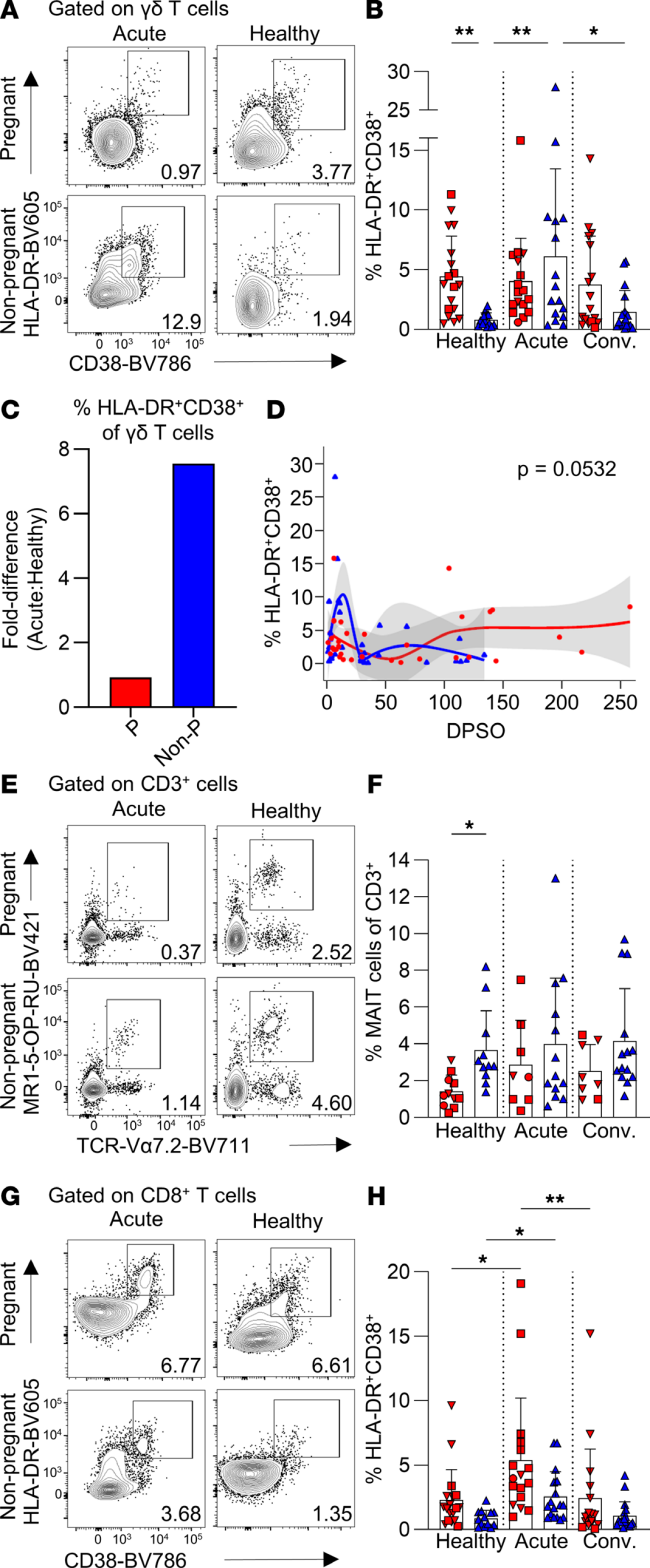
Differential γδ T cell activation dynamics in pregnant women with COVID-19. (**A**) CD3^+^γδTCR^+^ γδ T cell activation by HLA-DR and CD38 coexpression. (**B**) HLA-DR^+^CD38^+^ γδ T cell frequencies in healthy (P, *n* = 18; Non-P *n* = 13), acute (P, *n* = 17; Non-P, *n* = 16), and convalescent (P, *n* = 17; Non-P, *n* = 18) pregnant and nonpregnant women. (**C**) Fold difference in the mean frequency of HLA-DR^+^CD38^+^ γδ T cells between healthy and acute groups. (**D**) LOESS regression of HLA-DR^+^CD38^+^ γδ T cell frequencies and DPSO for pregnant and nonpregnant women with COVID-19. The 95% CI is shown in gray. (**E**) MAIT cells defined as MR1-5-OP-RU-tetramer^+^TCR-Vα7.2^+^. (**F**) Frequencies of MAIT cells in healthy (P, *n* = 11; Non-P, *n* = 11), acute (P, *n* = 8; Non-P, *n* = 13), and convalescent (P, *n* = 8; Non-P, *n* = 14) pregnant and nonpregnant women. (**G**) CD8^+^ T cell activation by HLA-DR and CD38 coexpression. (**H**) Frequencies of HLA-DR^+^CD38^+^ CD8^+^ T cells in healthy (P, *n* = 18; Non-P, *n* = 13), acute (P, *n* = 17; Non-P, *n* = 17), and convalescent (P, *n* = 17; Non-P, *n* = 18) pregnant and nonpregnant women. Means and SDs are shown in **B**, **F**, and **H**. Significance determined by Kruskal-Wallis test (**B**, **F**, and **H**) or Wald’s test (**D**). *P < 0.05; **P < 0.01.

Unconventional γδ T cells play an important role in antiviral responses to respiratory diseases, including influenza (50) and COVID-19 (51); however, their role and activation status in pregnant women with COVID-19 is undefined. Similar to our findings in NK cells, the proportion of activated HLA-DR^+^CD38^+^ γδ T cells was higher in healthy pregnant women compared with nonpregnant women (mean 4.4% and 0.8%, respectively; Figure 4, A and B), but became similar during acute COVID-19 (mean 4.0% and 6.1%, respectively; Figure 4B). Activation of γδ T cells in pregnant women remained stable during each disease state, whereas nonpregnant women with acute disease displayed a approximately 7.5-fold increase (Figure 4, B–D).

Further probing of HLA-DR^+^CD38^+^ γδ T cells revealed a similar distribution in activation of Vδ1 or Vδ2 subsets between pregnant and nonpregnant women (Supplemental Figure 3, B and C). However, during acute COVID-19 the Vδ1 subset comprised a larger proportion of activated γδ T cells, which decreased at convalescence for both pregnant and nonpregnant women (Supplemental Figure 3, C and D). As Vδ2 T cells are associated with cytotoxic activity (52, 53), the lower proportion observed in the blood during acute COVID-19 could arise from trafficking to the site of infection to perform effector functions, such as that observed in influenza virus studies (54, 55).

### Regulated MAIT and conventional T cell responses in pregnant women during COVID-19.

Defined by their ability to recognize MR1-5-OP-RU and biased expression of the Vα7.2 T cell receptor (TCR) chain, MAIT cells represent unconventional T cells with a role in antiviral immunity (51, 56–58). While MAIT cells characteristically recognize riboflavin metabolites, they can be activated as a first line of defense in a TCR-independent manner through cytokines, including IL-12 and IL-18 (56, 59). A decrease in peripheral blood MAIT cells was observed during COVID-19 (58). Our analysis revealed that healthy pregnant women had a lower frequency of MAIT cells compared with healthy nonpregnant women; however, no significant differences in MAIT cell frequencies in either group were observed during COVID-19 (Figure 4, E and F).

CD16^+^ patrolling monocytes were decreased in pregnant and nonpregnant women during acute COVID-19, as compared with their respective healthy and convalescent groups (Supplemental Figure 3, E and F), which is a prototypical response observed in COVID-19 (2).

Finally, classical αβ CD4^+^ and CD8^+^ T cell activation followed a similar trajectory in pregnant and nonpregnant women with COVID-19. CD8^+^ T cells followed a prototypical activation pattern during acute COVID-19, with a significant increase in HLA-DR^+^CD38^+^ CD8^+^ T cells and granzyme and perforin expression (Figure 4, G and H, and Supplemental Figure 3, G and H). No significant changes in CD4^+^ T cell activation, defined by HLA-DR and CD38 expression, were detected (Supplemental Figure 3, J–M); however, both pregnant and nonpregnant women had significant increases in polyfunctional CD4^+^ T cells expressing granzymes and perforin during acute COVID-19 (Supplemental Figure 3N).

Importantly, while key differences were observed in NK and γδ T cell activation, similar dynamics in CD4^+^ and CD8^+^ T cells suggests that TCR-mediated responses in the context of peptide-MHC presentation may not be affected by pregnancy.

### Immune cell activation within the placenta is similar in COVID-19 convalescent and healthy pregnancies.

The placenta forms the maternal-fetal interface that allows for exchange of nutrients and waste products. There is limited evidence that SARS-CoV-2 can cross the placenta (60, 61); however, antiviral immune responses could potentially alter the immune landscape in the placenta even after COVID-19 resolves. To assess for any changes in cellular immune components within placental tissue, flow cytometry was performed on placental single-cell suspensions from 9 COVID-19 convalescent and 6 SARS-CoV-2–unexposed pregnancies. While not statistically significant (*P* = 0.1520), we observed a trend toward lower MAIT cell frequencies in COVID-19 convalescent placentae compared with unexposed pregnancies (Supplemental Figure 4A). There was no difference in NK cell activation between COVID-19 and unexposed placenta samples (Supplemental Figure 4B), with a similar finding observed in CD56^bright^ and CD56^dim^ subsets (Supplemental Figure 4, C and D). Additionally, similar polyfunctionality of NK cells, determined by expression of intracellular granzymes and perforin, was observed between placentae from healthy and COVID-19 pregnancies (Supplemental Figure 4E). In contrast to observations in maternal blood at both acute and convalescent time points (Figure 3F), there were nearly equivalent proportions of CD56^bright^ and CD56^dim^ subsets comprising the placental NK cell population for healthy (mean 34.8% and 45.1%, respectively) and COVID-19 convalescent pregnancies (mean 41.2% and 33.9%) (Supplemental Figure 4G). There were significantly higher frequencies of CD56^bright^ NK cells in the placental tissue compared with matched maternal PBMCs (mean 38.8% and 13.4%, *P* = 0.0005; Supplemental Figure 4G), typically observed in healthy pregnancies (63). Activation of αβ CD4^+^ and CD8^+^ T cells and γδ T cells was similar between placentae from unexposed and COVID-19 pregnancies (Supplemental Figure 4, H–J). These findings indicate that immune cell activation between placentae from COVID-19 and healthy pregnancies are similar.

### Elevated levels of IL-8, IL-10, and IL-18 associated with healthy pregnancy.

**Figure 5:**
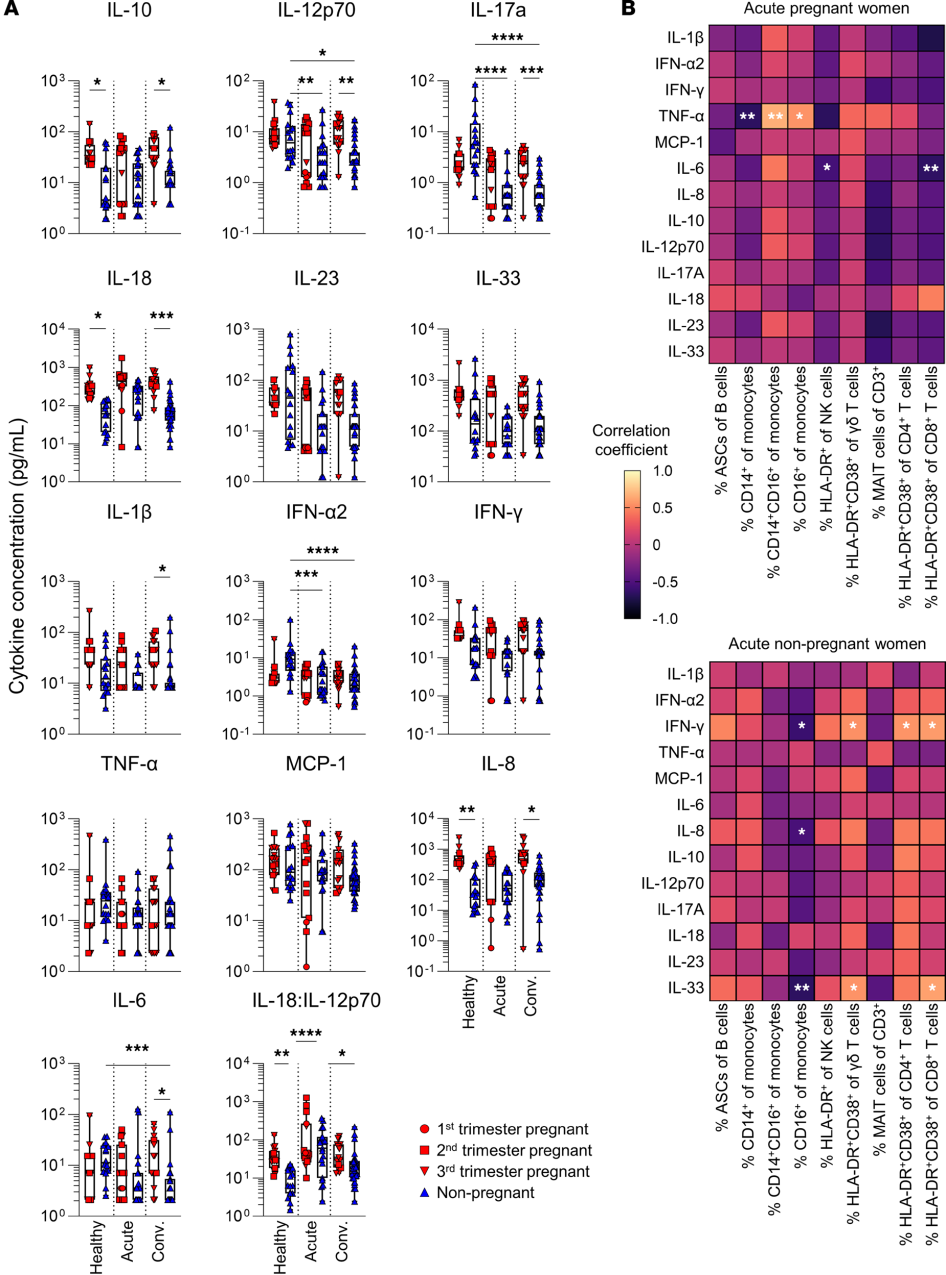
Cytokine and chemokine concentrations and proportions within blood plasma. (**A**) Concentrations of 13 cytokines/chemokines and IL-18/IL-12p70 ratio in pregnant and nonpregnant women who were healthy (P, *n* = 15; Non-P, *n* = 11) or had acute (P, *n* = 13; Non-P, *n* = 11) or convalescent (P, *n* = 14; Non-P, *n* = 26) COVID-19. Means and SDs are shown. Significance determined by Kruskal-Wallis test. (**B**) Heatmap depicting Spearman’s correlation coefficients for cytokine/chemokine concentrations against cellular immune parameters in acute COVID-19 pregnant (top) and nonpregnant (bottom) women. Significant correlations are depicted with an asterisk; *P* values are unadjusted. *P < 0.05; **P < 0.01; ***P < 0.001; ****P < 0.0001.

As dysregulation of inflammatory cytokines and chemokines is associated with severe COVID-19 (2, 63), we assessed cytokine/chemokine profiles to understand whether the inflammatory response during COVID-19 differed in pregnancy. As pregnant women are in a differential state of inflammation due to gestation, with relatively higher levels of IL-1β, IL-4, IL-5, and IL-10 (64), we hypothesized this could impact cytokine/chemokine levels during acute COVID-19. Elevated levels of IL-8, IL-10, and IL-18 were found in pregnant women in their healthy state, and these cytokine levels remained elevated during COVID-19 (Figure 5A and Supplemental Figure 5). Interestingly, IL-17a and IFN-α2 were significantly elevated in healthy nonpregnant women compared with acute or convalescent counterparts, but not this was not observed in pregnant women (Figure 5A), and the kinetics of IL-23 differed between pregnant and nonpregnant women with COVID-19 (Supplemental Figure 5). As higher IL-18 concentration was associated with healthy pregnancy, and IL-18 function is dependent on IL-12 (65), we assessed the IL-18/IL-12p70 ratio. Healthy pregnant women had a higher IL-18/IL-12p70 ratio than nonpregnant women, indicating more IL-18 availability, likely resulting in the Th2 bias expected during healthy pregnancy. Overall, differences in cytokines were associated with pregnancy status, with no differences observed between pregnant and nonpregnant women with acute COVID-19.

To gain insights into immune profiles associated with SARS-CoV-2 infection during pregnancy, we correlated cytokine levels and immune cell populations during acute COVID-19 in pregnant and nonpregnant individuals. TNF-α positively correlated with inflammatory CD14^+^CD16^+^ and patrolling CD16^+^ monocytes in pregnant women with acute COVID-19, while negatively correlating with classical CD14^+^ monocytes and activated NK cells (Figure 5B). Interestingly, these correlations were not observed in nonpregnant individuals, which may be due to pregnant women shifting away from Th1 immunity, leading to increased levels of inflammatory CD14^+^CD16^+^ and patrolling CD16^+^ monocytes, which are the main drivers of TNF-α production (66, 67). Rather, IFN-γ concentrations in nonpregnant women positively correlated with activated CD4^+^, CD8^+^, and γδ T cells, while negatively correlating with patrolling CD16^+^ monocytes (Figure 5B). This is not unexpected, as nonpregnant women can sufficiently mount a coordinated Th1 response to SARS-CoV-2, whereas pregnant women need to maintain a more regulated inflammatory milieu.

Moreover, similar levels of cytokines were detected in cord blood from healthy and COVID-19 convalescent pregnancies (Supplemental Figure 6). Overall, our cytokine data suggest that pregnant and nonpregnant women have a differential inflammatory state when healthy, whereas cytokine profiles become more similar during acute COVID-19.

### Immune network analysis reveals a comprehensive map of immune responses to COVID-19 in pregnancy.

**Figure 6:**
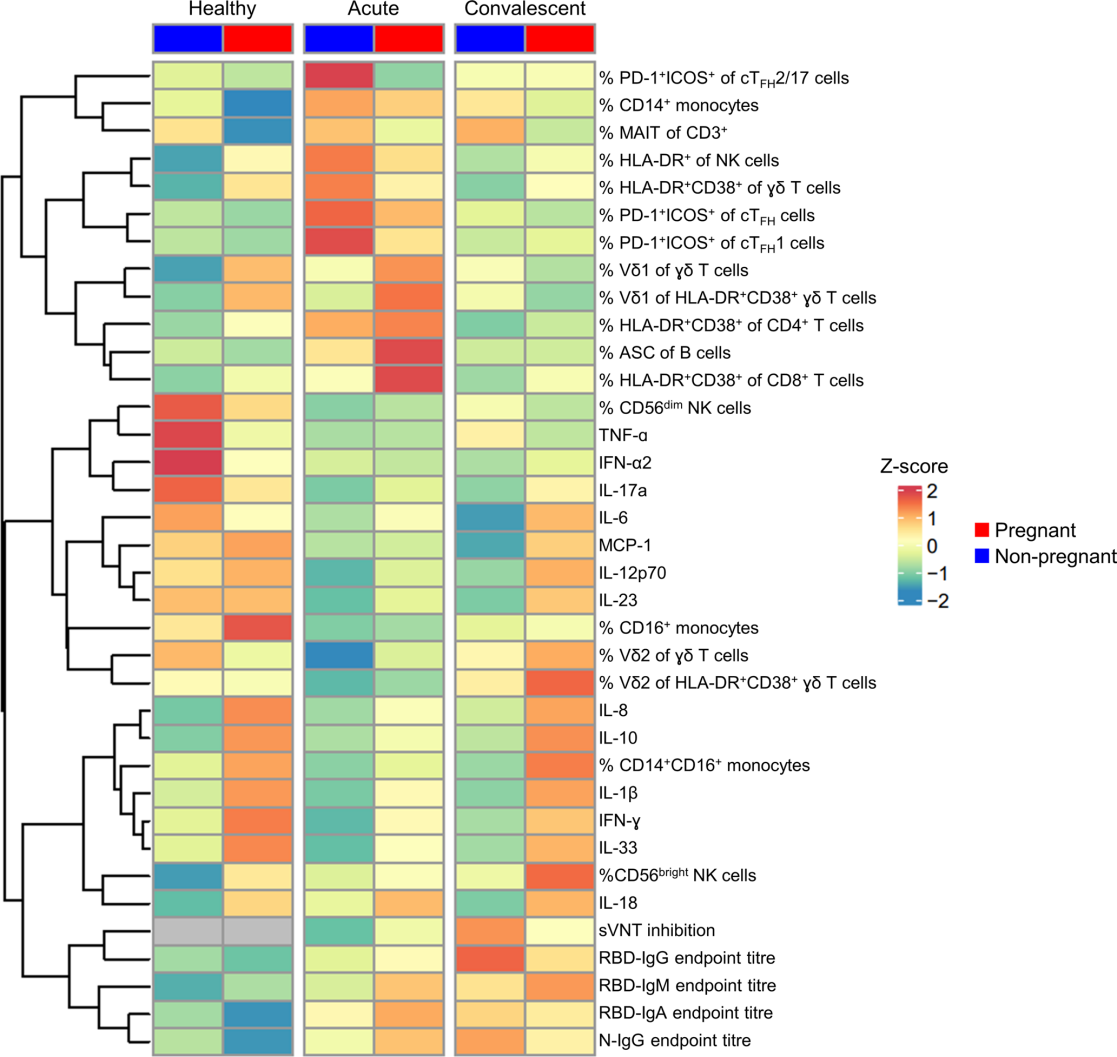
Summary data of key differences in immune parameters between pregnant and nonpregnant women. Heatmap depicting the mean of 36 selected immune parameters for healthy, acute, and convalescent pregnant and nonpregnant women. *Z*-score values are shown.

**Figure 7:**
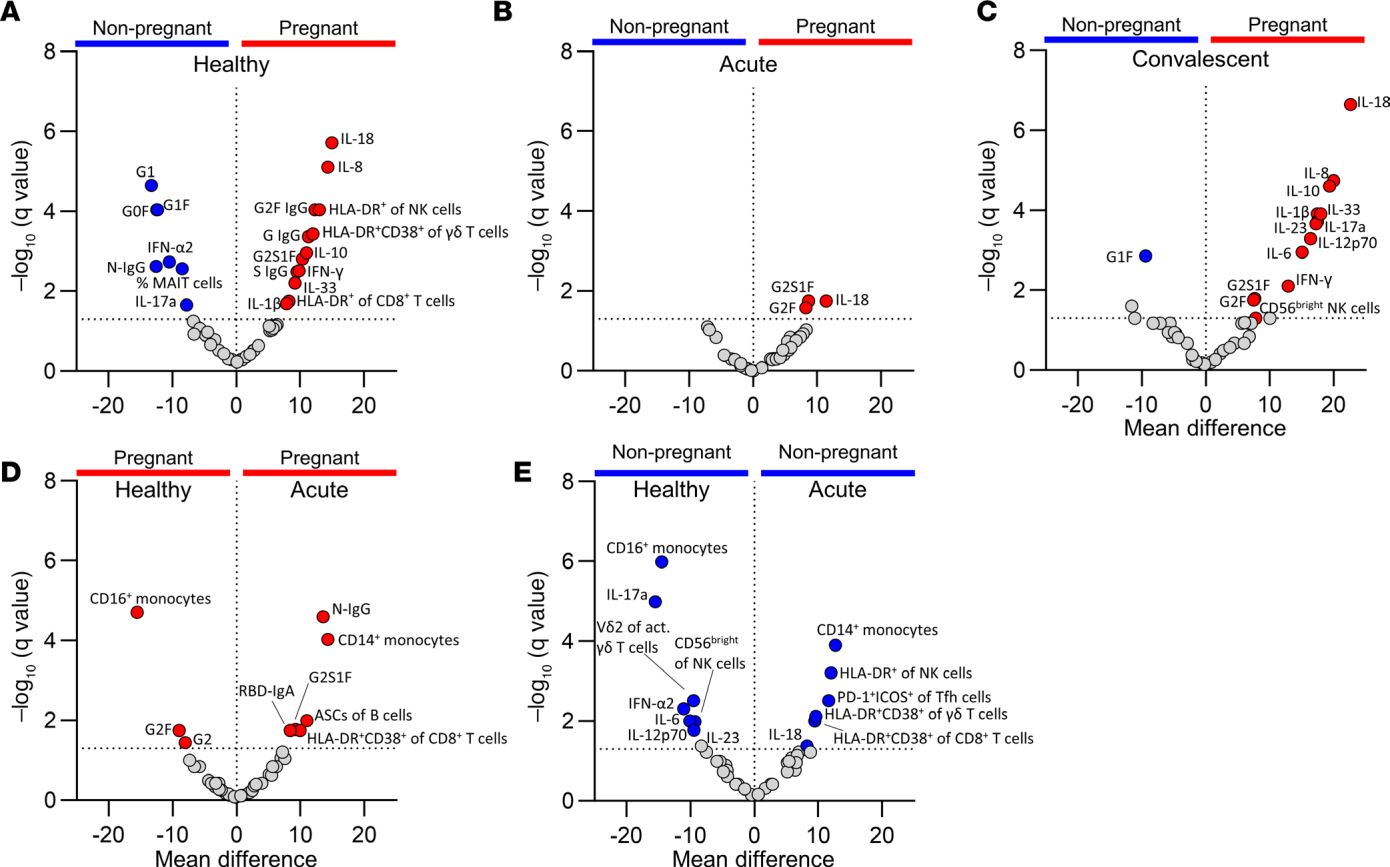
Factors driving differences between pregnant and nonpregnant women diminish during acute COVID-19. (**A**–**E**) Volcano plots of 47 selected cellular and humoral immune parameters between (**A**) healthy or (**B**) acute or (**C**) convalescent pregnant and nonpregnant women, and between healthy and acute (**D**) pregnant or (**E**) nonpregnant women.

We comprehensively analyzed 217 immunological parameters between pregnant and nonpregnant women, including antibodies, cellular subsets, and cytokines/chemokines and revealed distinct profiles between pregnant and nonpregnant women (Figure 6). While striking differences were found between pregnant and nonpregnant women in the healthy state and convalescent COVID-19, immune responses were more comparable during acute COVID-19 (Figure 7, A–E), reflecting the preactivated and inflamed state in pregnant women. For example, in the healthy state, pregnant women displayed profound upregulation of HLA-DR^+^ NK cells, HLA-DR^+^CD38^+^ γδ T cells, IL-8, IL-10, and IL-18 (Figure 7A). These differences became less apparent during acute COVID-19, when pregnant women appeared to have prototypical antiviral immunity (Figure 7B). To assess differences occurring due to pregnancy or COVID-19, we also compared healthy and acute COVID-19 pregnant women (Figure 7D). When comparing healthy and acute data in pregnant and nonpregnant women, there were overall fewer significantly different features, suggesting that the observed differences are linked to pregnancy status rather than COVID-19. However, both pregnant and nonpregnant women had an increase in HLA-DR^+^CD38^+^ CD8^+^ T cells and CD14^+^ classical monocytes during acute COVID-19, as compared with their respective healthy group (Figure 7, D and E). Collectively, the immune responses in pregnant women with acute COVID-19 closely resembled those in acute COVID-19 nonpregnant women, although the dynamics of these responses differ due to inherent immune adaptations during pregnancy. Conversely, at convalescence, hyperactivation of immune responses in pregnant women was again observed, especially with respect to cytokine production (IL-1β, IL-6, IL-8, IL-10, IL-12, IL-17a, IL-18, IL-23, and IL-33) and CD56^bright^ NK cells (Figure 7C). Additionally, as there was a level of heterogeneity in this study regarding pregnancy trimester and DPSO at which blood samples were collected, we used a mixed effect multiple regression to assess the significance of these continuous variables. This analysis showed that the variability in time since infection (DPSO) and week of pregnancy did not impact the comparisons of immune responses to SARS-CoV-2 infection, with the exception of MCP-1 being positively associated with the week of pregnancy (Supplemental Table 6).

## Discussion

Pregnant women are a vulnerable group for SARS-CoV-2 infection (19), as published reports correlated pregnancy with an increased risk of ICU admission, invasive ventilation, ECMO (19, 20), death, sepsis, mechanical ventilation, shock, acute renal failure, thromboembolic disease (21), and hypertensive complications (22). However, not all studies reveal strong correlations between pregnancy and COVID-19 severity or prolonged disease complications (24), and not at the level that occurred during the 2009 H1N1 pandemic (27). As more epidemiological data emerge regarding COVID-19 pregnancies, it is essential to comprehensively define immune responses to understand whether SARS-CoV-2 immunity is prototypical, as observed in mild to moderate COVID-19 in nonpregnant individuals (2–4, 15, 16), or conversely, is characterized by immune perturbations similar to those observed during severe COVID-19 (2, 18). Our study fills this knowledge gap and provides the first comprehensive map to our knowledge of immunological responses in pregnant women during acute and convalescent phases of SARS-CoV-2 infection.

Our in-depth analysis of RBD-specific IgG, IgM, and IgA, N-specific IgG, neutralizing activity, and multidimensional systems serology parameters together with ASCs and Tfh cells showed comparable antibody features between pregnant and nonpregnant women during SARS-CoV-2 infection. We also provided evidence of RBD- and N-specific IgG antibodies found in cord blood of convalescent pregnancies. Our data validate previous studies showing generation of SARS-CoV-2–specific antibodies in pregnant women following infection (30–32).

Comparison of cellular immunity in pregnant and nonpregnant women with COVID-19 revealed prototypical activation patterns of classical αβ CD4^+^ and CD8^+^ T cells, while NK and γδ T cell activation was similar during acute infection, indicating a typical COVID-19 innate response, as observed by others (2, 68). Higher frequencies of activated NK and γδ T cells during healthy pregnancy seems to have prevented any further activation during acute COVID-19. Additionally, a lower frequency of circulating MAIT cells was observed in healthy pregnant women, although we did not observe any decrease in their frequency during acute COVID-19, which has been associated with COVID-19 disease severity (57, 58). Differential NK cell activation has been reported in healthy pregnant women and during influenza infection (47, 69); however, γδ T and MAIT cell activation following viral infection during pregnancy is understudied. It was recently reported that healthy pregnant women in their second or third trimester display higher frequencies of a CD56^+^ γδ T cell subset and higher cytotoxic potential reflected by expression of CD107a, compared with nonpregnant women (70). We found higher frequencies of HLA-DR–expressing γδ T cells, suggesting an important role for activated γδ T cells in normal pregnancy. Alongside higher CD56^+^ γδ T cells, pregnant women also have higher levels of CD56^+^ and CD69^+^ MAIT cells, compared with nonpregnant women (71). Additionally, women who were previously pregnant had substantially increased frequencies of PD-1^+^Vδ2^+^ γδ T cells compared with nulliparous women or women with recurrent pregnancy loss (72). Furthermore, enrichment of Vδ1^+^ and HLA-DR^+^ γδ T cells was observed in the decidua during early pregnancy (73), and increased frequencies of MAIT cells in the intervillous blood within healthy-term placenta (74). These reports suggest an important role for unconventional T cells in pregnancy.

IL-8, IL-10, and IL-18 were increased in healthy pregnant women and remained elevated during COVID-19. Interestingly, IL-18–dependent MAIT cell activation was reported following influenza infection (56), suggesting a role for IL-18 in mediating MAIT cell activation during pregnancy, as it was found at increased concentrations in our analysis. Others have found an increase in IL-8 and IL-10 in SARS-CoV-2–infected pregnant women as compared with their healthy counterparts (75). While we did not observe these differences between healthy and acute pregnant women, there were higher levels when compared with nonpregnant women. The higher IL-18/IL-12p70 ratio observed in healthy pregnant women in our study and others (76) indicates a skewing toward Th2 immunity (65), which could impact the establishment of an antiviral immune response. Similarly, it has been reported that pregnant and nonpregnant women with COVID-19 share similar cytokine and chemokine profiles, with the exception of eotaxin and GRO-a (77), while key differences between COVID-19 and healthy pregnant women were found in IL-12p70, MIP-1β, and RANTES (78). Additionally, Pinheiro et al. (64) showed that pregnant women have higher levels of regulatory cytokines, including IL-1β, IL-4, IL-5, and IL-10, compared with nonpregnant women. Overall, our data set similarly identifies a Th2 cytokine response associated with pregnancy, with fewer differences observed between pregnant and nonpregnant women with acute COVID-19.

Our analyses of immune cells within placentae from COVID-19 or healthy pregnancies revealed comparable levels NK and T cell activation. The similarities between healthy and COVID-19 placental immune cell activation might be due to COVID-19 placentae being from convalescent time points. However, a recent study examining placental histological features from women acutely infected with SARS-CoV-2 at the time of birth found no significant differences in histopathology, suggesting that COVID-19 does not directly cause pathology in this tissue (79).

Collectively, we provide evidence that antibody and adaptive cellular compartments are similar between unvaccinated pregnant and nonpregnant women with acute or convalescent COVID-19; however, the innate compartment, namely NK and γδ T cell activation and inflammation, is perturbed during pregnancy. These findings provide key insights for further studies on immune responses in pregnancy, and will help inform patient management and education of COVID-19 during pregnancy.

### Limitations of our study.

It should be noted that pregnancy presents a dynamic immune state that is time dependent. Our COVID-19 pregnancy cohort consisted of pregnant women within the first, second, or third trimester of pregnancy. These factors may have implications for the findings presented here. Further studies are needed to specifically focus on SARS-CoV-2 immune responses within each trimester of pregnancy and the postpartum period to improve our understanding of the immune responses. Additionally, the timing of sample collection relative to disease onset was variable between individuals. While individuals could be allocated into acute (1–20 DPSO) or convalescent (21–258 DPSO) groups based on a typical timeframe of disease recovery, and allow for kinetic analyses, the spread in timing could have an impact on the results presented here. Future work should involve a cohort with samples at key disease and gestational time points for a more uniform data set.

## Methods

### Study participants.

One hundred and nineteen individuals were included in this study, from which 158 blood samples were collected for immune analyses (Supplemental Tables 1 and 2). This study analyzed non–SARS-CoV-2–vaccinated self-reported females of reproductive age who were recruited into larger patient cohorts. Blood samples were collected from 23 pregnant and 33 nonpregnant women with acute or convalescent COVID-19 between March 2020 and March 2021 via the Mercy Hospital for Women, Royal Women’s Hospital, Royal Melbourne Hospital, Austin Hospital, Alfred Hospital, Children’s Hospital Los Angeles, or University of Melbourne. Twenty-one healthy pregnant donors were sampled via the Mercy Hospital for Women and University of Melbourne. Blood samples were collected from 42 healthy nonpregnant donors via University of Melbourne or the Australian Red Cross LifeBlood (West Melbourne, Australia). Individuals with acute COVID-19 had blood collected between 1 and 17 DPSO and were symptomatic at the time of sampling, with the exception of 1 asymptomatic pregnant individual sampled 1 day after testing PCR-positive. Convalescent individuals were recovered from symptoms and where relevant, discharged from hospital prior to sample collection. This study was a part of a larger study to understand immune responses to COVID-19 and immune perturbation during severe COVID-19.

### Blood and placenta sample collection and processing.

Blood samples were collected in heparinized tubes. Plasma was obtained by centrifugation of heparinized blood tubes at 300*g* for 10 minutes. PBMCs were isolated by density-gradient centrifugation (Ficoll-Paque, Cytiva). Placentae were obtained within 15 minutes of birth. Placental lobules (cotyledons) were obtained from multiple locations on the maternal surface. The basal plate and chorionic surface were removed from the cotyledons, and villous tissue obtained from the middle cross section. Placental tissue was washed 3 times in PBS before being submerged and kept at 4°C until processing (within 24 hours). Placentae were mechanically dissociated and subjected to enzymatic digestion with 2 mg/mL Collagenase D (Roche) in RPMI 1640 with 0.2 mg/mL DNase I (Roche), 1 mM HEPES, penicillin, and streptomycin for 1 hour at 37°C. Cells were filtered through 70-μm strainers and red blood cells (RBC) lysed using 0.168 M NH_4_Cl, 0.01 mM EDTA, and 12 mM NaHCO_3_ in ddH_2_O. Isolated PBMCs and placental single-cell suspensions were cryopreserved in fetal calf serum/10% DMSO.

### Flow cytometry of whole blood, PBMCs, and placentae.

Cellular immunity was assessed using fresh whole blood, PBMCs, or placental single-cell suspensions, as described previously (3). Four antibody panels were used to determine (a) activation of monocytes, T, B, NK, and γδ T cells, (b) Tfh cells and ASCs, (c) polyfunctionality of T and NK cells, and (d) MAIT/γδ T cell phenotypes (Supplemental Figures 7 and 8). Cells were stained, RBC lysed (whole blood), fixed in 1% paraformaldehyde, or stained intracellularly using eBioscience Foxp3/Transcription Factor Staining Buffer (Thermo Fisher Scientific), as described previously (3). Samples were acquired on an LSR Fortessa (BD Biosciences) and analyzed using FlowJo v10 software.

### SARS-CoV-2 RBD and N ELISA.

ELISAs to detect RBD- or N-specific IgG, IgM, and IgA were performed as described previously (2, 11, 16). Briefly, Nunc MaxiSorp flat-bottom 96-well plates (Thermo Fisher Scientific) were coated with antigen (2 μg/mL) and blocked with PBS (with 1% BSA, w/v) for at least 1 hour. Donor plasma was added in log_0.5_ dilutions and incubated for 2 hours at room temperature. Bound antibodies were detected using either HRP-conjugated anti–human IgG or IgM with TMB substrate, or alkaline phosphatase–conjugated rat anti–human IgA with *p*-nitrophenyl phosphate (disodium salt) substrate. Peroxidase reactions were stopped using 1 M H_3_PO_4_. Optical densities were determined using a Multiskan plate reader (Labsystems). Endpoint titers were determined by interpolation of a sigmoidal curve fit (*R*^2^ values >0.95; GraphPad Prism 9), as the reciprocal dilution of plasma that produced greater than 15% (for IgA and IgG) or greater than 30% (IgM) absorbance of the positive control at 1:31.6 (IgG and IgM) or 1:10 dilutions (IgA). Seroconversion was defined as a titer greater than the mean plus 2 SD of non–COVID-19 control plasma samples.

### Antibody avidity ELISA.

RBD-specific IgG and IgM avidity was measured by urea-mediated dissociation ELISA, as described previously (16). ELISA was performed as above, with the modification of washing wells and incubating with 6 M urea for 15 minutes after plasma incubation. Bound antibodies were detected using HRP-conjugated anti-human IgG or anti-human IgM antibody as above. The amount (%) of antibody remaining was determined by comparing the total area of the antibody titration curve (across 4 dilutions) in the presence and absence of urea treatment and is expressed as the avidity index.

### sNVT.

A SARS-CoV-2 sVNT kit (GenScript) was used to detect antibodies that block the interaction between the SARS-CoV-2 spike protein and ACE2, as described previously (16). HRP-conjugated recombinant SARS-CoV-2 RBD fragment bound to neutralizing antibodies, preventing capture by the human ACE2 protein in the well, which was subsequently removed in the following wash step. Substrate was added and incubated for 20 minutes at room temperature and results measured by spectrophotometry. Color intensity was inversely dependent on the titer of anti–SARS-CoV-2 neutralizing antibodies.

### Microneutralization test.

Microneutralization activity was determined as described previously (80). SARS-CoV-2 isolate CoV/Australia/VIC01/2020 (81) was propagated in Vero cells. Heat-inactivated plasma (56°C for 30 minutes) was serially diluted and serum/virus mixtures assessed for residual virus infectivity in quadruplicate wells of Vero cells incubated in serum-free media containing 1 μg/mL TPCK trypsin at 37°C and 5% CO_2_. Viral cytopathic effect was measured on day 5. Neutralizing antibody titers were calculated using the Reed-Muench method (80).

### Total IgG glycosylation.

Total IgG glycosylation was analyzed as described previously using capillary electrophoresis (82). Melon gel IgG purification resin was used to purify total IgG from plasma according to the manufacturer’s protocol (Thermo Fisher Scientific). N-linked glycans on purified IgG was analyzed using LabChip GXII Touch Microchip-CE platform per the manufacturer’s protocol (PerkinElmer).

### Coupling of carboxylated beads.

A custom multiplex bead array was designed (Supplemental Table 3) and coupled with SARS-CoV-1 and SARS-CoV-2 spike-1 (stem, Sino Biological), spike-2 (head, ACRO Biosystems), RBD (BEI Resources), and N (ACRO Biosystems), as described previously (45). In addition, SARS-CoV-2 spike trimers (provided in-house) and SARS-CoV-2 spike trimers (BPS Bioscience) were included. Tetanus toxoid (Sigma-Aldrich), influenza hemagglutinin (H1Cal2009, Sino Biological), and SIV-gp120 (Sino Biological) were included as positive and negative control antigens, respectively. Antigens were covalently coupled to magnetic carboxylated beads (Bio-Rad) using a 2-step carbodiimide reaction and blocked with 0.1% BSA, before being resuspended and stored in PBS with 0.05% sodium azide until use.

### Luminex bead-based multiplex assay.

A custom multiplex assay was used to investigate isotypes and subclasses of SARS-CoV-1– and SARS-CoV-2–specific antibodies in plasma samples (45). Briefly, 20 μL of working bead mixture and 20 μL of diluted plasma (final dilution 1:200) were added per well and incubated overnight at 4°C on a shaker. Fourteen detectors were used to assess pathogen-specific antibodies. Single-step detection was done using phycoerythrin-conjugated (PE-conjugated) mouse anti–human pan-IgG, IgG1-4, and IgA1-2 (Southern Biotech; 1.3 μg/mL, 25 μL/well). For the detection of FcγR binding, soluble recombinant FcγR dimers, which come in higher (FcγRIIa-H131 and FcγRIIIa-V158) or lower affinity (FcγRIIa-R131, FcγRIIb, and FcγRIIIa-F158; 1.3 μg/mL, 25 μL/well; provided in-house and as a gift from Bruce D. Wines, Melbourne, Australia), were first added to the beads, washed, and followed by addition of streptavidin-PE (SA-PE). For detection of IgM, biotinylated mouse anti–human IgM (mAb MT22, Mabtech; 1.3 μg/mL, 25 μL/well) was first added to beads, washed, followed by SA-PE. Assays were performed in duplicate and read on Flexmap 3D (Luminex).

### Data normalization for multiplex analysis.

Tetanus, H1Cal2009, BSA, and SIV control antigens were removed from the analysis. Low signal features were removed when the 75th percentile response for the feature was lower than the 75th percentile of the BSA positive control. Right shifting was performed on each feature (detector-antigen pair) individually if it contained any negative values, by adding the minimum value for that feature back to all samples within that feature. Right-shifted data were log-transformed to achieve normal distribution using the following equation, where *x* is the right-shifted data and *y* is the right-shifted log-transformed data: *y* = log_10_(*x* + 1). Data were further normalized by mean centering and variance scaling each feature using the *z* score function in MATLAB (MathWorks) in the subsequent multivariate analyses.

### Multivariable methods for identification of the key antibody features.

A least absolute shrinkage and selection operator (LASSO) penalized logistic regression model was used to determine the minimal set of features needed to predict pregnancy status during acute and convalescent COVID-19 (83). The LASSO shrinks data toward a model with fewer parameters and identifies which antibody features best discriminate between 2 groups. The frequency of selected features in resampling was considered as the criterion of variable importance (83). The feature selection stability was defined as the proportion of times a feature was picked in the selected set of important features, when the model was repeatedly fitted to 1000 resampled subsets of data. Inner cross validations (CVs) ranging from 4-fold to 10-fold were performed for each of the resampled data sets. Following model prediction, 10-fold CV was selected due to its consistency.

### Principal component analysis.

PCA was performed in the Eigenvectors PLS toolbox (Eigenvector Research, Inc.) in MATLAB. PCA is an unsupervised technique to visualize the variance between samples based on all measured features and allows for dimensionality reduction (84). Each antibody feature is assigned a loading that in linear combinations creates a principal component (PC). Loadings and PCs describe the maximum amount of variance in the data set. Two-dimensional score plots were generated to visually assess separation between groups using their individual response measurements expressed through the PCs. The percentage variance described by each PC describes the amount of variance in antibody response explained by that respective PC (45).

### Cytokine measurements.

Plasma levels of IL-1β, IFN-α2, IFN-γ, TNF-α, MCP-1, IL-6, IL-8, IL-10, IL-12p70, IL-17A, IL-18, IL-23, and IL-33 were measured using the LEGENDplex Human Inflammation Panel-1 kit (BioLegend). Plasma was diluted 1:2. The assay was performed according to the manufacturer’s instructions. Samples were acquired on a FACSCanto II (BD Biosciences) and analyzed with QOGNIT LEGENDplex software.

### Statistics.

Data and statistical analyses were performed in GraphPad Prism (v9) unless otherwise stated. LOESS regression plots were made in R studio (v4) using the ggplot2 package (85). PESTLE and SPICE software (v6.1) were used for analysis of granzyme/perforin coexpression; a permutations test determined statistical significance (86). Correlation analyses were performed using nonparametric Spearman’s *r*. For statistical analyses, *P* values less than 0.05 were considered significant.

### Multivariate analysis.

To assess the significance of infection status, pregnancy status, week of pregnancy, and time since infection, we used a mixed effect multiple regression. We treated both pregnancy status and infection status as binary variables. We asked whether (a) time since infection and pregnancy status were predictors within COVID-19 groups, (b) week of pregnancy and infection status were predictors within pregnant groups, and (c) week of pregnancy and time of infection were predictors within COVID-19 pregnant groups.

Significance was determined by Wald’s test, which can be obtained based on the standard error of the estimated parameters in the model. The model was fitted using the *nlme* library in R (v4.04) (87).

### Study approval and ethics statement.

Experiments conformed to the Declaration of Helsinki Principles and the Australian National Health and Medical Research Council Code of Practice. Written informed consents were obtained from all donors prior to the study. The study was approved by the Alfred Hospital (no. 182/20), Melbourne Health (HREC/66341/MH-2020 and HREC/17/MH/53), Austin Health (HREC/63201/Austin-2020), Monash Health (HREC/15/MonH/64), Mercy Health (R14/25 and R04/29), Children’s Hospital Los Angeles (CHLA-20-00124), St. Jude Children’s Research Hospital (19-0009), Australian Red Cross Lifeblood (2015#08), and University of Melbourne (nos. 1749349, 2056901, 1443540, 2056761, 1955465, 2020-20782-12450-1, and 2021-13973-14410-3) human research ethics committees (HRECS). All external primary HRECs (outside the University of Melbourne) were registered and ratified by the University of Melbourne Ethics Committee with the associated University of Melbourne project ID nos. 13344, 20782, 1749349, 2056901, and 1443540.

### Data availability.

Data underlying figures and supplemental figures, and FACS source files are available from authors upon request. Raw FACS data are shown in the manuscript.

## Author contributions

KK and LCR supervised the study. KK, LCR, JRH, BYC, LK, HFK, SN, and AWC designed experiments. JRH, BYC, LK, KJS, TD, ERH, THON, HFK, SN, XJ, LFA, LH, WZ, CEVSS, JAN, HXT, and LCR performed and analyzed experiments. TD, HAM, LH, AR, and MPD analyzed data. FA, FK, AKW, and PMH provided reagents, including SARS-CoV-2 spike trimers provided by AKW and recombinant FcγR dimers provided by PMH. KP, JSYL, JJ, EKA, KMW, JAJ, AKW, GP, SW, JA, IT, JTD, ACC, SYCT, KB, DAW, JHM, PGT, PSP, FJ, NEH, OCS, JAT, CLG, CLW, SJK, and ML recruited patient cohorts and provided clinical data. JRH, SN, KS, DIG, AWC, SJK, ML, LCR, and KK provided intellectual input into study design and data interpretation. JRH, LCR, and KK wrote the manuscript. All authors reviewed and approved the manuscript.

## Supplementary Material

Supplemental data

## Version 1 04/10/2023

Electronic publication
