# Supplementary material for: Immune profiling of SARS-CoV-2 infection during pregnancy reveals NK cell and γδ T cell perturbations
Source: JCI Insight. 2023 Apr 10;8(7):e167157. doi: 10.1172/jci.insight.167157 (PMC10132165; doi:10.1172/jci.insight.167157)
Supplement: Supplemental data [file jciinsight-8-167157-s204.pdf]

Supplementary Information

Immune profiling of SARS-CoV-2 infection during pregnancy reveals NK cell and γδ T cell perturbations

Jennifer R Habel[1], Brendon Y Chua[1,2], Lukasz Kedzierski[1,3], Kevin J Selva[1], Timon Damelang[1], Ebene R Haycroft[1], Thi HO Nguyen[1], Hui-Fern Koay[1], Suellen Nicholson[4], Hayley McQuilten[1], Xiaoxiao Jia[1], Lilith F Allen[1], Luca Hensen[1], Wuji Zhang[1], Carolien E van de Sandt[1], Jessica A Neil[1], Katherine Pragastis[5], Jillian SY Lau[5,6], Jaycee Jumarang[7], E. Kaitlynn Allen[8], Fatima Amanant[9,10], Florian Krammer[9], Kathleen Wragg[1], Jennifer A Juno[1], Adam K Wheatley[1, 11], Hyon-Xhi Tan[1], Gabrielle Pell[12], Susan Walker[12], Jennifer Audsley[13], Arnold Reynaldi[14], Irani Thevarajan[13, 15], Justin Denholm[13, 15], Kanta Subbarao[1, 16], Miles P Davenport[14], P. Mark Hogarth[17-19], Dale I Godfrey[1], Allen C Cheng[20, 21], Steven YC Tong[15, 22], Katherine Bond[1, 23], Deborah A Williamson[1, 23], James McMahon[5], Paul G Thomas[8], Pia S Pannaraj[7,24], Fiona James[25], Natasha E Holmes[25-28], Olivia C Smibert[25, 29, 30], Jason A Trubiano[28-31], Claire L Gordon[1, 25], Amy W Chung[1], Clare L Whitehead[32,33], Stephen J Kent[1,11,34], Martha Lappas[12, 35], Louise C Rowntree[1#] and Katherine Kedzierska[1,2#]

Supplementary Figure 1. SARS-CoV-2-specific antibodies in pregnant and non-pregnant women, and maternal-cord dyads. (A) RBD-specific IgG, IgM and IgA plasma dilution curves for healthy (P n=10, Non-P n=27), acute COVID-19 (P n=13, Non-P n=11) or convalescent COVID-19 (P n=15, Non-P n=33) pregnant and non-pregnant donors. (B) Geometric mean titres of neutralizing antibodies in plasma from pregnant and non-pregnant healthy (n=15 and 15), acute (n= 13 and 10) and convalescent donors (n=13 and 13). Black dashed line indicates detection limit, orange dashed lines indicates seroconversion cut-off. (C) Avidity of RBD-specific IgG and IgM antibodies in pregnant (n=7) and non-pregnant (n=12) COVID-19 donors performed across sequential bleeds. (D) Proportions of pregnant and non-pregnant donors who seroconverted according to RBD-specific IgG titres. (E) Log_{10} RBD- and N-specific IgG and RBD-specific IgM and IgA titres in cord blood from healthy (n=5) and COVID-19 (n=9) pregnancies. Orange dashed lines indicate seroconversion cut-off based on healthy cord blood titres (mean plus two standard deviations), cut-off could not be calculated for IgA due to lack of variance. (F) RBD-specific and N-specific IgG, and sVNT percentage inhibition in matched maternal-cord pairs (n=9). (G) Antibody end point titres of RBD-specific IgM and IgA in matched maternal-cord pairs (n=9). (H) Mean fluorescence intensity of spike-head-specific or spike-stalk-specific IgG2 in healthy (P=10, Non-P=15), acute COVID-19 (P=13, Non-P=11) and convalescent COVID-19 (P=9, Non-P=10) pregnant and non-pregnant donors. *$p<0.05$, **$p<0.01$, ***$p<0.001$, ****$p<0.0001$.

Supplementary Figure 2. IgG glycosylation. (A) Relative abundance of different glycosylation patterns on total IgG from pregnant and non-pregnant healthy (n=15 and 14), acute (n=13 and 7), convalescent (n=12 and 11) COVID-19 donors, plus healthy cord blood (n=10) and COVID-19 exposed cord blood (n=6). (B) Proportions of IgG glycotypes in healthy or acute/convalescent pregnant or non-pregnant women. *$p<0.05$, **$p<0.01$, ***$p<0.001$, ****$p<0.0001$.

Supplementary Figure 3. CD4 and CD8 T cell activation and monocyte subsets. (A) Proportions of $CD56^{bright}CD16^-$, and $CD56^{dim}CD16^+$ in $HLA\text{-}DR^+$ NK cells. (B) Vδ1, Vδ2 and non- Vδ1/2 subsetting of γδ T-cells. (C) Proportions of Vδ1, Vδ2 and non-Vδ1/2 within the $HLA\text{-}DR^+CD38^+$ γδ T-cell population in pregnant and non-pregnant women who were healthy (P n=11, Non-P n=11) or had acute (P n=8, Non-P n=13) or convalescent (P n=7, Non-P n=15) COVID-19. (D) LOESS regression of the proportions of Vδ1 (left) or Vδ2 (right) T-cells within the activated γδ T-cell population and DPSO for pregnant (n=15) and non-pregnant (n=28) women with COVID-19. (E) Differential gating of $CD14^+CD16^-$ classical, $CD14^+CD16^+$

inflammatory and CD14$^-$CD16$^+$ patrolling monocytes. (F) Proportions of classical (blue), inflammatory (yellow-green) and patrolling (green) monocytes. (G) Fold-difference in the mean frequency of HLA-DR$^+$CD38$^+$ CD8$^+$ T cells from healthy to acute COVID-19 for pregnant and non-pregnant donors. (H) LOESS regression of the frequency of HLA-DR$^+$CD38$^+$ CD8$^+$ T cells and DPSO for pregnant (n=36) and non-pregnant women (n=36) with COVID-19. (I) Proportions of granzymes A, B, K and M and perforin expression in HLA-DR$^+$CD38$^+$ CD8$^+$ T cells in pregnant and non-pregnant healthy (n=15 and 11), acute (n=16 and 15), convalescent (n=14 and 15) COVID-19 women. (J) CD4$^+$ T cell activation was determined by HLA-DR and CD38 expression. (K) Frequencies of HLA-DR$^+$CD38$^+$ CD4$^+$ T cells in pregnant and non-pregnant women who were healthy (P n=18, Non-P n=13), acute (P n=17, Non-P n=17) or convalescent (P n=16, Non-P n=19) from COVID-19. (L) Fold-difference in the mean frequency of HLA-DR$^+$CD38$^+$ CD4$^+$ T cells from healthy to acute COVID-19 for pregnant and non-pregnant donors. (M) Correlation between the frequency of HLA-DR$^+$CD38$^+$ CD4$^+$ T cells and DPSO for pregnant and non-pregnant women with COVID-19. (N) Proportions of granzymes A, B, K and M and perforin expression in HLA-DR$^+$CD38$^+$ CD4$^+$ T cells.

Supplementary Figure 4. (A) Frequencies of MAIT cells in unexposed (n=7) and COVID-19 convalescent (n=8) placenta. (B-D) Frequencies of HLA-DR$^+$ total (B), CD56^{bright} (C) and CD56^{dim} (D) NK cells in placenta tissue from unexposed (n=6) and COVID-19 (n=8) pregnancies. (E) Proportions of granzymes A, B, K and M and perforin expression in total (top), CD56^{dim} (middle) and CD56^{bright} (bottom) NK cells. (F) Proportions of CD56^{bright}CD16$^-$, CD56^{dim}CD16$^+$ and non-CD56$^{bright/dim}$ (CD56^{dim}CD16$^-$) NK cells within unexposed and COVID-19 placentae. (G) Frequencies of CD56^{bright} NK cells in matched maternal peripheral PBMC-placenta pairs. COVID-19 and unexposed pregnancies are combined in the Wilcoxon statistical test to determine statistical significance. (H-J) Frequencies of HLA-DR$^+$CD38$^+$ (H) CD4$^+$, (I) CD8$^+$ and (J) γδ T cells.

Supplementary Figure 5. Cytokine/chemokine kinetics in COVID-19 donors. LOESS regressions of cytokine or chemokine concentration and DPSO for pregnant and non-pregnant COVID-19 donors.

Supplementary Figure 6. Cytokines in unexposed and COVID-19 pregnancy cord blood. Concentrations of thirteen cytokines detected in cord blood from unexposed (n=10) and COVID-19 (n=8) pregnancies.

Supplementary Figure 7. (A) Gating strategy for monocytes, T, B, NK and γδ T cells and (B) T_{FH} and ASC cell activation (Supp Table 4). The PD-1/ICOS FACS plot shown in the gating strategy is the same as the acute non-pregnant representative plot shown in Fig 1J.

Supplementary Figure 8. (A) Gating strategy for the cytotoxicity profiles of CD4$^+$ and CD8$^+$ T cells and NK cells expressing intracellular granzymes A, B, K and M and perforin (Supp Table 5). (B) Gating strategy for activation and phenotypes of MAIT and γδ T cells (Supp Table 5).

Supplementary Table 1. Cohort summary

	Healthy		Acute		Convalescent	
	Pregnant	Non-pregnant	Pregnant	Non-pregnant	Pregnant	Non-pregnant
Number of donors	21	42	12	17	14	25
Sample size	21	42	17	20	16	42
Age, median (range)	33 (24-42)	31.5 (18-48)	31 (20-36)	27 (21-47)	31.5 (25-40)	28 (21-49)
Female (%)	100%	100%	100%	100%	100%	100%

Days post symptom onset at collection, median (range)	N/A	N/A	7 (1-17)	6 (1-13)	107 (21-258)	68 (28-208)
Location during acute disease						
Intensive care unit (ICU)	N/A	N/A	0	2	1	0
Hospital ward	N/A	N/A	6	8	4	4
Home/Outpatient	N/A	N/A	6	7	9	22
Week gestation at sample collection, median (range)	39 (20-41)		24 (5-39)		37 (22-42)	
Fetal sex (% Female)	57%		71%		54%	

Supplementary Table 2. Cohort clinical data

						Days post symptom onset at					Week of gestation at			
Donor Code	Age	Pregnant (Y/N)	Disease	Fetal sex	Recruitment Site	Visit 1	Visit 2	Visit 3	Visit 4	Days in hospital	Visit 1	Visit 2	Visit 3	Visit 4
NP-C-01	29	N	COVID-19		Austin Health	8	11			2				
NP-C-02	22	N	COVID-19		Austin Health	7	12			2				
NP-C-03	47	N	COVID-19		Austin Health	9				27				
NP-C-04	27	N	COVID-19		Austin Health	13				1				
NP-C-05	30	N	COVID-19		Austin Health	7	110			2				
NP-C-06	25	N	COVID-19		Austin Health	2				6				
NP-C-07	31	N	COVID-19		Austin Health	10	11	113		2				
NP-C-08	25	N	COVID-19		Alfred Hospital	1				6				
NP-C-09	30	N	COVID-19		Melbourne Health	110								
NP-C-10	43	N	COVID-19		Melbourne Health	114								
NP-C-11	35	N	COVID-19		Melbourne Health	119								
NP-C-12	24	N	COVID-19		Melbourne Health	85								
NP-C-13	31	N	COVID-19		Melbourne Health	121								
NP-C-14	27	N	COVID-19		Melbourne Health	134								
NP-C-15	21	N	COVID-19		University of Melbourne	8	28	59	126					
NP-C-16	22	N	COVID-19		University of Melbourne	32	72	205						
NP-C-17	49	N	COVID-19		University of Melbourne	29	61	143						
NP-C-18	22	N	COVID-19		University of Melbourne	47	68	131						
NP-C-19	49	N	COVID-19		University of Melbourne	44	208							
NP-C-20	28	N	COVID-19		University of Melbourne	49	144							
NP-C-21	49	N	COVID-19		University of Melbourne	45	87	136						
NP-C-22	24	N	COVID-19		University of Melbourne	56	80	131						
NP-C-23	34	N	COVID-19		University of Melbourne	45	119							
NP-C-24	32	N	COVID-19		University of Melbourne	45								
NP-C-25	38	N	COVID-19		Monash Health	2	30							
NP-C-26	23	N	COVID-19		Monash Health	1	68							
NP-C-27	22	N	COVID-19		Monash Health	2	44							
NP-C-28	27	N	COVID-19		Monash Health	4	35							
NP-C-29	29	N	COVID-19		Monash Health	32								
NP-C-30	28	N	COVID-19		Monash Health	3	30							
NP-C-31	25	N	COVID-19		Monash Health	4								
NP-C-32	25	N	COVID-19		Monash Health	2	29							
NP-C-33	27	N	COVID-19		Monash Health	29								
NP-C-34	32	N	COVID-19		Monash Health	3	31							
P-C-01	31	Y	COVID-19	M	Austin Health	11	12			2	24	24		
P-C-02	36	Y	COVID-19	F	Austin Health	12	17			6	19	20		
P-C-03	32	Y	COVID-19	F	Austin Health	6				1	35			
P-C-04	30	Y	COVID-19	F	Mercy Hospital	30				4	39			
P-C-05	34	Y	COVID-19	F	Mercy Hospital	55				1	42			
P-C-06	35	Y	COVID-19	M	Mercy Hospital	104					39			
P-C-07	36	Y	COVID-19	F	Mercy Hospital	141				6	38			
P-C-08	28	Y	COVID-19	M	Mercy Hospital	115					40			
P-C-09	32	Y	COVID-19	F	Mercy Hospital	144					40			
P-C-10	32	Y	COVID-19	F	Mercy Hospital	217					36			
P-C-11	31	Y	COVID-19	M	Mercy Hospital	198					38			
P-C-12	35	Y	COVID-19	M	Mercy Hospital	258					40			
P-C-13	33	Y	COVID-19	F	Melbourne Health	6	8			5	28	28		
P-C-14	25	Y	COVID-19	F	Melbourne Health	1	3	5	21	5	25	25	26	28
P-C-15	29	Y	COVID-19	NA	University of Melbourne	10	79	139			16	26	34	
P-C-16	27	Y	COVID-19	NA	University of Melbourne	10					21			
P-C-17[#]	40	Y	COVID-19	M/F	University of Melbourne	32				17	29			
P-C-18	30	Y	COVID-19	M	University of Melbourne	63	110				22	29		
P-C-19	26	Y	COVID-19	F	Melbourne Health	6				5	21			
P-C-20	33	Y	COVID-19	M	CHLA	4	68			20	29			
P-C-21	31	Y	COVID-19	F	CHLA	0*				39				
P-C-22	20	Y	COVID-19	NA	CHLA	14				5				
P-C-23	23	Y	COVID-19	M	CHLA	6				15				
NP-H-01	20	N	Healthy		Red Cross Lifeblood									
NP-H-02	21	N	Healthy		University of Melbourne									
NP-H-03	33	N	Healthy		University of Melbourne									
NP-H-04	47	N	Healthy		University of Melbourne									
NP-H-05	36	N	Healthy		University of Melbourne									
NP-H-06	41	N	Healthy		University of Melbourne									
NP-H-07	25	N	Healthy		University of Melbourne									
NP-H-08	32	N	Healthy		University of Melbourne									
NP-H-09	36	N	Healthy		University of Melbourne									
NP-H-10	28	N	Healthy		University of Melbourne									
NP-H-11	32	N	Healthy		University of Melbourne									
NP-H-12	40	N	Healthy		University of Melbourne									
NP-H-13	41	N	Healthy		University of Melbourne									
NP-H-14	37	N	Healthy		University of Melbourne									
NP-H-15	46	N	Healthy		University of Melbourne									
NP-H-16	28	N	Healthy		University of Melbourne									
NP-H-17	24	N	Healthy		University of Melbourne									
NP-H-18	36	N	Healthy		University of Melbourne									
NP-H-19	38	N	Healthy		University of Melbourne									

NP-H-20	43	N	Healthy		University of Melbourne	
NP-H-21	22	N	Healthy		University of Melbourne	
NP-H-22	21	N	Healthy		University of Melbourne	
NP-H-23	21	N	Healthy		University of Melbourne	
NP-H-24	35	N	Healthy		University of Melbourne	
NP-H-25	25	N	Healthy		University of Melbourne	
NP-H-26	25	N	Healthy		University of Melbourne	
NP-H-27	29	N	Healthy		University of Melbourne	
NP-H-28	32	N	Healthy		University of Melbourne	
NP-H-29	33	N	Healthy		University of Melbourne	
NP-H-30	48	N	Healthy		University of Melbourne	
NP-H-31	26	N	Healthy		University of Melbourne	
NP-H-32	28	N	Healthy		University of Melbourne	
NP-H-33	33	N	Healthy		University of Melbourne	
NP-H-34	32	N	Healthy		University of Melbourne	
NP-H-35	24	N	Healthy		University of Melbourne	
NP-H-36	37	N	Healthy		University of Melbourne	
NP-H-37	31	N	Healthy		University of Melbourne	
NP-H-38	20	N	Healthy		University of Melbourne	
NP-H-39	18	N	Healthy		University of Melbourne	
NP-H-40	23	N	Healthy		University of Melbourne	
NP-H-41	27	N	Healthy		University of Melbourne	
NP-H-42	31	N	Healthy		University of Melbourne	
P-H-01	36	Y	Healthy	M	Mercy Hospital	39
P-H-02	28	Y	Healthy	M	Mercy Hospital	39
P-H-03	32	Y	Healthy	F	Mercy Hospital	39
P-H-04	36	Y	Healthy	F	Mercy Hospital	39
P-H-05	33	Y	Healthy	F	Mercy Hospital	41
P-H-06	32	Y	Healthy	M	Mercy Hospital	38
P-H-07	39	Y	Healthy	F	Mercy Hospital	40
P-H-08	35	Y	Healthy	F	Mercy Hospital	39
P-H-09	31	Y	Healthy	M	Mercy Hospital	39
P-H-10	24	Y	Healthy	F	Mercy Hospital	38
P-H-11	42	Y	Healthy	M	Mercy Hospital	39
P-H-12	31	Y	Healthy	M	Mercy Hospital	40
P-H-13	32	Y	Healthy	M	Mercy Hospital	21
P-H-14	37	Y	Healthy	F	Mercy Hospital	24
P-H-15	29	Y	Healthy	F	Mercy Hospital	25
P-H-16	33	Y	Healthy	F	Mercy Hospital	22
P-H-17	33	Y	Healthy	M	Mercy Hospital	20
P-H-18	39	Y	Healthy	F	Mercy Hospital	38
P-H-19	35	Y	Healthy	F	University of Melbourne	26
P-H-20	25	Y	Healthy	M	Mercy Hospital	38
P-H-21	27	Y	Healthy	F	Mercy Hospital	39

CHLA: Children's Hospital Los Angeles
NA: data not available
Twin pregnancy
* Asymptomatic individual. Day post-PCR+ result used instead

Supplementary Table 3. Panel design of the multiplex bead array assay

Pathogens	Proteins	Isotypes and FcɣR bindings
SARS-CoV-2	RBD	IgG
	S1	IgG1
	S2	IgG2
	Trimeric S	IgG3
	NP	IgG4
SARS-CoV-1	S1	IgA1
	Trimeric S	IgA2
	NP	IgM
C. Tetani	Tetanus Toxin	FcɣRIIaH
Influenza A/Cali/07/2009 (H1N1)	Hemagglutinin	FcɣRIIaR
		FcɣRIIb
		FcɣRIIIaV
		FcɣRIIIaF

Supplementary Table 4. Statistics for key features in multiplex PCA loading plots.

Multiplex feature	P value	Median (range)	Median (range)
		Acute pregnant	Acute non-pregnant
IgA2 SARS2 S2	0.1633	2993 (37.13-481144)	272.1 (28.13-141975)
IgA2 SARS1 Trimer S	0.3378	28 (7-1108)	22 (2.5-53)
IgA1 SARS1 Trimer S	0.2342	247 (0-8367)	159.5 (0.5-885.5)
IgA1 SARS1 N	0.494	2430 (83-68816)	1843 (75.5-15935)
IgG2 SARS1 Trimer S	0.0397	201 (188-464.5)	190 (179-242)
IgM SARS2 Trimer S	0.5309	70051 (5885-169762)	25738 (7044-195754)
IgM SARS2 RBD	0.3031	16924 (2443-138031)	7960 (1005-158256)
IgG4 SARS1 Trimer S	0.6183	115 (99-133.5)	117.5 (99-131)
IgG2 SARS2 RBD	0.0312	31.5 (10.5-111)	13 (3-99.5)
IgG3 SARS2 S1	0.6085	2262 (2221-9067)	2243 (2222-15284)
FcgRIIIaV SARS2 S1	0.54	101.5 (43.5-13661)	74.5 (47-1308)
IgG1 SARS2 Trimer S	0.8646	16827 (584-152455)	3075 (797-85784)
FcgRIIb SARS2 Trimer S	0.6802	25.5 (9-14067)	63 (4.5-13375)
IgG3 SARS1 Trimer S	0.0488	2253 (2222-2357)	2231 (2205-2289)
		Convalescent pregnant	Convalescent non-pregnant
FcgRIIIaV SARS2 N	0.6038	726 (0-15394)	3575 (205-5825)
FcgRIIIaV SARS2 RBD	0.447	353 (33-6762)	788.8 (54-3460)
FcgRIIb SARS2 N	0.0681	54.1 (14.6-6762)	19.6 (4.1-157.1)
Pan IgG SARS2 Trimer S	0.2775	21411 (1962-131326)	46061 (6938-85283)
IgG1 SARS1 N	0.4002	10930 (318-141845)	32462 (90.5-81227)
IgG3 SARS1 N	0.549	4222 (2263-54998)	3392 (2513-9511)
IgG2 SARS2 RBD	0.033	58 (16-602.5)	39 (15-244)
FcgRIIb SARS1 Trimer S	0.7345	20 (3-108)	17 (7.5-32)
FcgRIIaR SARS2 RBD	0.1275	31 (12-1816)	23.25 (6-75.5)
IgA2 SARS2 S2	0.0535	789.1 (361.6-471634)	256.9 (40.13-66732)

Supplementary Table 5. Mixed effect modelling statistical output

Parameter	Fixed effects	Value	Standard Error	Degrees of freedom	t-value	p-value
% ASCs of B cells	Day post symptom onset	-0.0040302	0.001844	7	-2.1855564	0.0651
	Week of pregnancy	-0.007747	0.0137364	7	-0.5639783	0.5904
% HLA-DR^{+} of NK cells	Day post symptom onset	0.000616	0.00145719	7	0.4227602	0.6852
	Week of pregnancy	0.0033184	0.01116068	7	0.2973306	0.7748
% HLA-DR^{+}CD38^{+} of γδ T cells	Day post symptom onset	0.0004226	0.002018	7	0.2094128	0.8401
	Week of pregnancy	0.0019951	0.0156195	7	0.1277287	0.902
IL-1β	Day post symptom onset	0.0011033	0.00133637	7	0.825581	0.4363
	Week of pregnancy	0.004569	0.01006304	7	0.454037	0.6635
IFN-γ	Day post symptom onset	-0.0017473	0.0021178	7	-0.825021	0.4366
	Week of pregnancy	0.0125504	0.1497	7	0.83837	0.4295
TNF-α	Day post symptom onset	0.0004457	0.002286	7	0.194971	0.851
	Week of pregnancy	-0.0076357	0.0138492	7	-0.551346	0.5985
MCP-1	Day post symptom onset	-0.0047773	0.0020562	7	-2.32342	0.0531
	Week of pregnancy	0.0451879	0.0148078	7	3.051618	0.0185*
IL-6	Day post symptom onset	-0.0012312	0.0020017	7	-0.615856	0.558
	Week of pregnancy	0.0131544	0.0139719	7	0.9414869	0.3778
IL-8	Day post symptom onset	-0.0020817	0.003189	7	-0.6527536	0.5348
	Week of pregnancy	0.0446053	0.0251255	7	1.7752984	0.1191
IL-10	Day post symptom onset	0.0025689	0.001304	7	1.970094	0.0895
	Week of pregnancy	0.0066419	0.0119992	7	0.553525	0.5971
IL-12p70	Day post symptom onset	0.002431	0.00112235	7	2.16601	0.067
	Week of pregnancy	0.0044054	0.01063177	7	0.41436	0.691
IL-17a	Day post symptom onset	0.00110968	0.00147625	7	0.7516874	0.4767
	Week of pregnancy	0.00747841	0.0112267	7	0.6661267	0.5267
IL-18	Day post symptom onset	-0.0016375	0.00168444	7	-0.972147	0.3634
	Week of pregnancy	0.0210935	0.01197256	7	1.76182	0.1215
IL-23	Day post symptom onset	0.0019456	0.00193387	7	1.006043	0.3479
	Week of pregnancy	0.0050249	0.01407421	7	0.357027	0.7316
% HLA-DR^{+}CD38^{+} of CD4+ T cells	Day post symptom onset	0.0000527	0.0022	7	0.0239515	0.9816
	Week of pregnancy	-0.0183947	0.0168629	7	-1.0908396	0.3115
% HLA-DR^{+}CD38^{+} of CD8+ T cells	Day post symptom onset	-0.0011148	0.0020135	7	-0.5536783	0.597
	Week of pregnancy	-0.0078303	0.0163859	7	-0.4778659	0.6473
Surrogate virus neutralization test (sVNT)	Day post symptom onset	-0.005847	0.016878	7	-0.3464112	0.7392
	Week of pregnancy	-0.061441	0.135932	7	-0.4519946	0.6649
RBD-IgG titre	Day post symptom onset	0.00643	0.0044508	7	0.14447	0.8892
	Week of pregnancy	-0.0109745	0.0336954	7	-0.325696	0.7542
N-IgG titre	Day post symptom onset	-0.004142	0.0027072	6	-1.530096	0.1769
	Week of pregnancy	0.000212	0.0206825	6	0.010256	0.9921

*p<0.05

Supplementary Table 6. Flow cytometry panels

Panel	Ab/Dye/Tetramer	Clone	Fluorochrome	Vendor	Catalogue number	Dilution
Lymphocyte phenotyping panel	CD71	M-A712	BV421	BD	562995	1:50
	CD19	SJ25C1	BV510	BD	562947	1:200
	HLA-DR	L243	BV605	Biolegend	307640	1:50
	CD4	SK3	BV650	BD	563875	1:200
	CD27	L128	BV711	BD	563167	1:200
	CD38	HIT2	BV786	BD	563964	1:200
	CD56	MEM-188	APC	Biolegend	304610	1:50
	CD16	3G8	AF700	Biolegend	302026	1:50
	CD14	MφP9	APC-H7	BD	560180	1:50
	CD45RA	HI100	FITC	BD	555488	1:50
	CD8	SK1	PerCP-Cy5.5	BD	565310	1:200
	γδTCR	11F2	PE	BD	347907	1:100
	CD3	UCHT1	PE-CF594	BD	562280	1:800
	PD-1	EH12.1	PE-Cy7	BD	561272	1:100
ASC/T_{FH} panel	CXCR5	RF8B2	BV421	BD	562747	1:50
	CD19	SJ25C1	BV510	BD	562947	1:200
	CD24	ML5	BV605	BD	562788	1:100
	CCR6	11A9	BV650	BD	563922	1:600
	CD20	2H7	BV711	BD	563126	1:200
	CD38	HIT2	BV786	BD	563964	1:200
	CXCR3	1C6	APC	BD	550967	1:25
	CD27	M-T271	AF700	BD	560611	1:100
	CD4	RPA-T4	APC-H7	BD	560158	1:150
	CD8	HIT8a	FITC	BD	555634	1:200
	CD45	2D1	PerCP-Cy5.5	BD	340953	1:50
	ICOS	DX29	PE	BD	557802	1:10
	CD3	UCHT1	PE-CF594	BD	562280	1:800
	PD-1	EH12.1	PE-Cy7	BD	561272	1:100
Granzyme and Perforin panel	CD8	RPA-T8	BV421	Biolegend	301036	1:200
	CD3	OKT3	BV510	Biolegend	317332	1:200
	HLA-DR	L243	BV605	Biolegend	307640	1:100
	CD4	SK3	BV650	BD	563875	1:200
	CD25	2A3	BV711	BD	563159	1:100
	CD38	HIT2	BV786	BD	563964	1:200
	Granzyme M	4B2G4	eFlour660	Invitrogen	50-9774-42	1:50
	Granzyme B	GB11	AF700	BD	560213	1:50
	CD14	MφP9	APC-H7	BD	560180	1:100
	CD19	SJ25C1	APC-H7	BD	560252	1:100
	CD56	B159	BB515	BD	564488	1:25
	Granzyme K	G3H69	eFlour710	Invitrogen	46-8897-42	1:50
	Granzyme A	CB9	PE	Invitrogen	12-9177-42	1:50
	FoxP3	236A/E7	PE-CF594	BD	563955	1:100
	Perforin	B-D48	PE-Cy7	Biolegend	353316	1:10
MAIT and γδ T cell panel	CD16	3G8	BUV395	BD	563785	1:100
	MR1-5-OP-RU tetramer	N/A	Streptavidin-BV421	Biolegend (streptavidin)	405225 (streptavidin)	1 ug/mL
	Live/Dead	N/A	Aqua	Invitrogen	L34957	1:800
	HLA-DR	L243	BV605	Biolegend	307640	1:100
	Vα7.2	3C10	BV711	Biolegend	351732	1:100
	Vδ2	B6	BV711	Biolegend	331412	1:300
	CD38	HIT2	BV786	BD	563964	1:200
	CD27	M-T271	AF700	BD	560611	1:50
	CD69	FN50	APC-Cy7	Biolegend	310914	1:100
	Vδ1	TS8.2	FITC	Invitrogen	TCR2730	1:100

Vγ9	B3	PE-Cy5	Biolegend	331323	1:150
γδTCR	B1	PE-Cy7	Biolegend	331222	1:50
CD94	HP-3D9	BUV395	BD	743954	1:200
CD4	SK3	BUV496	BD	612936	1:100
CD3	UCHT1	BUV737	BD	612750	1:100

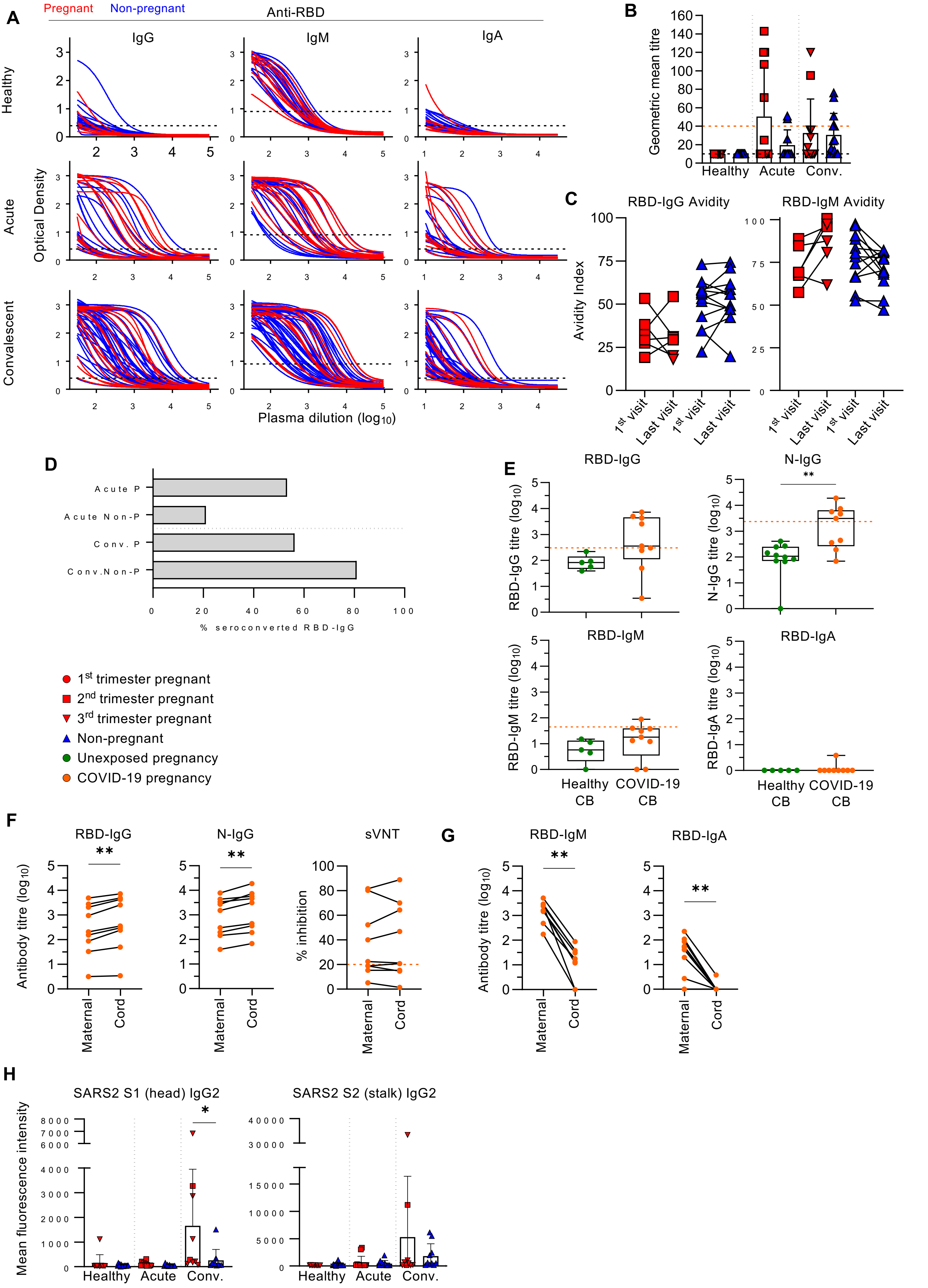

Supplementary Figure 1

Habel et al.

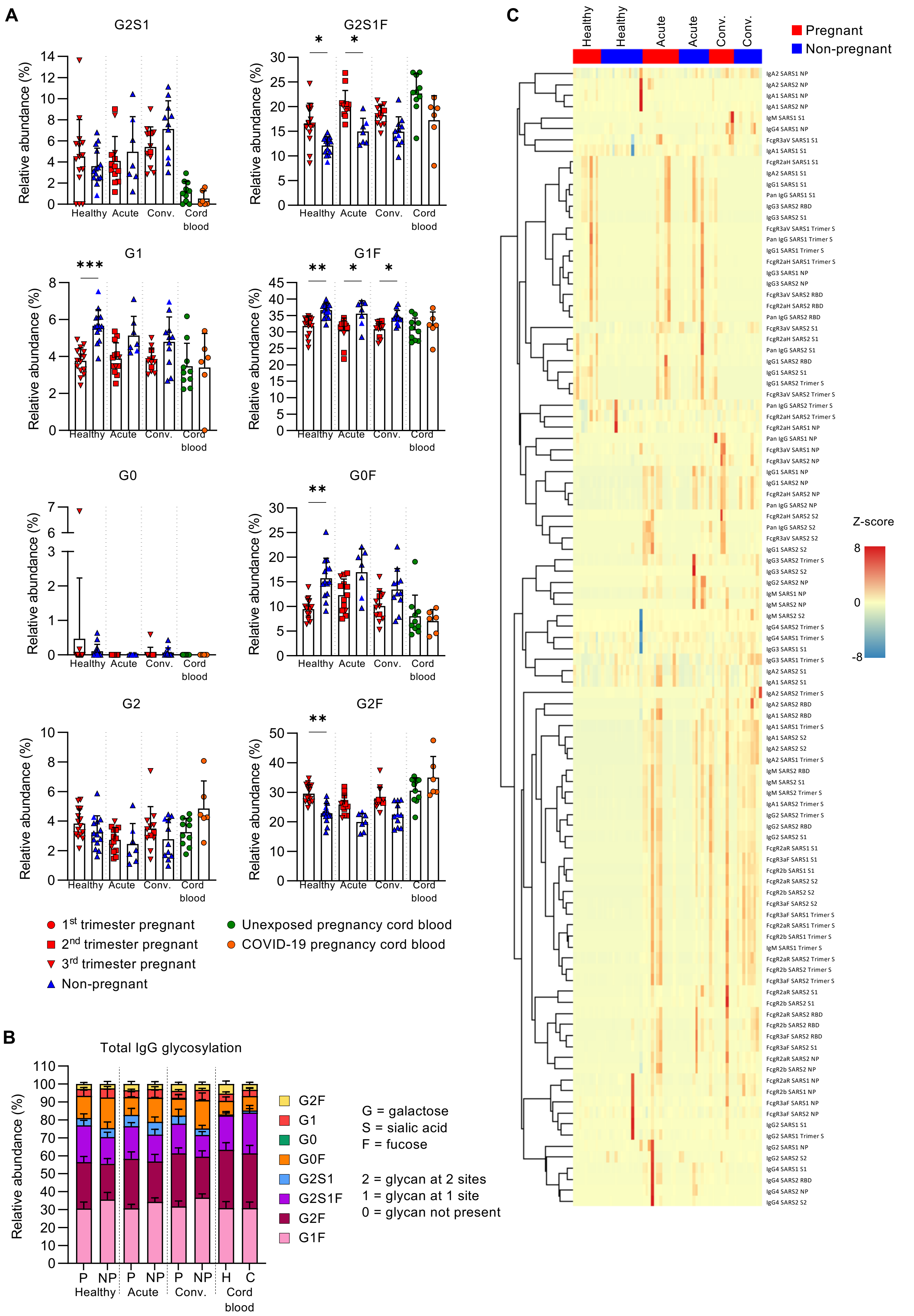

Supplementary Figure 2

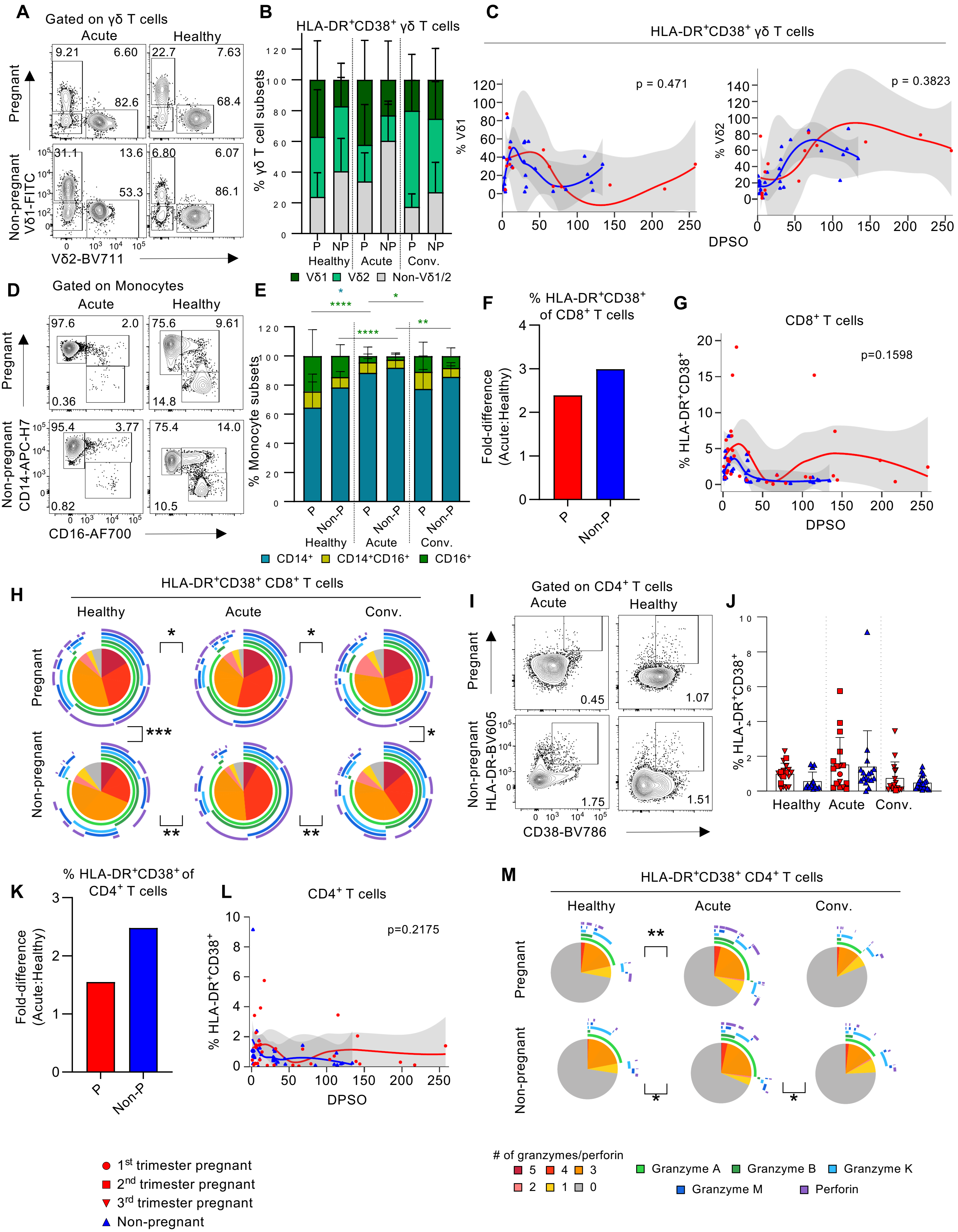

Supplementary Figure 3

Habel et al.

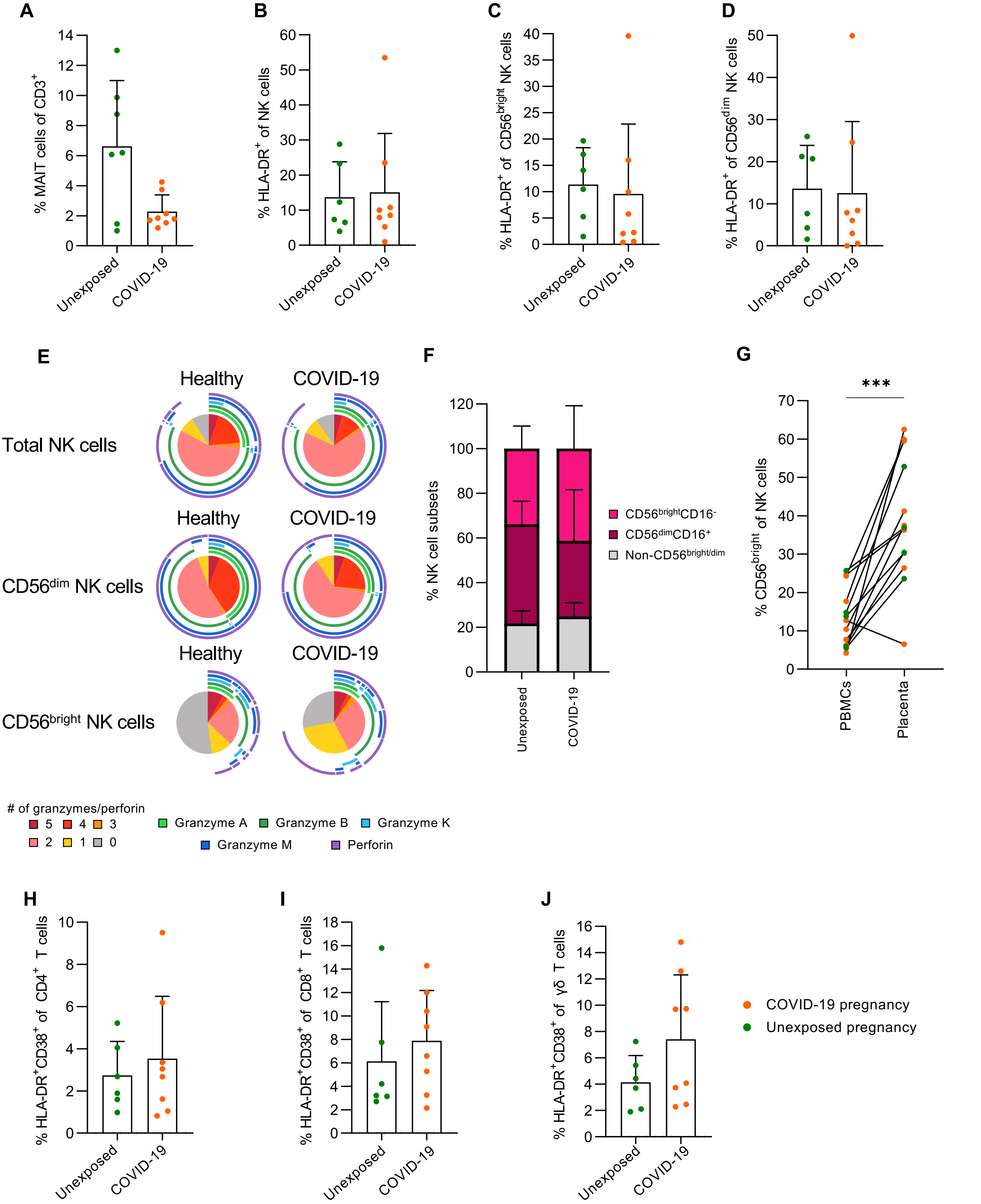

Supplementary Figure 4

Habel et al.

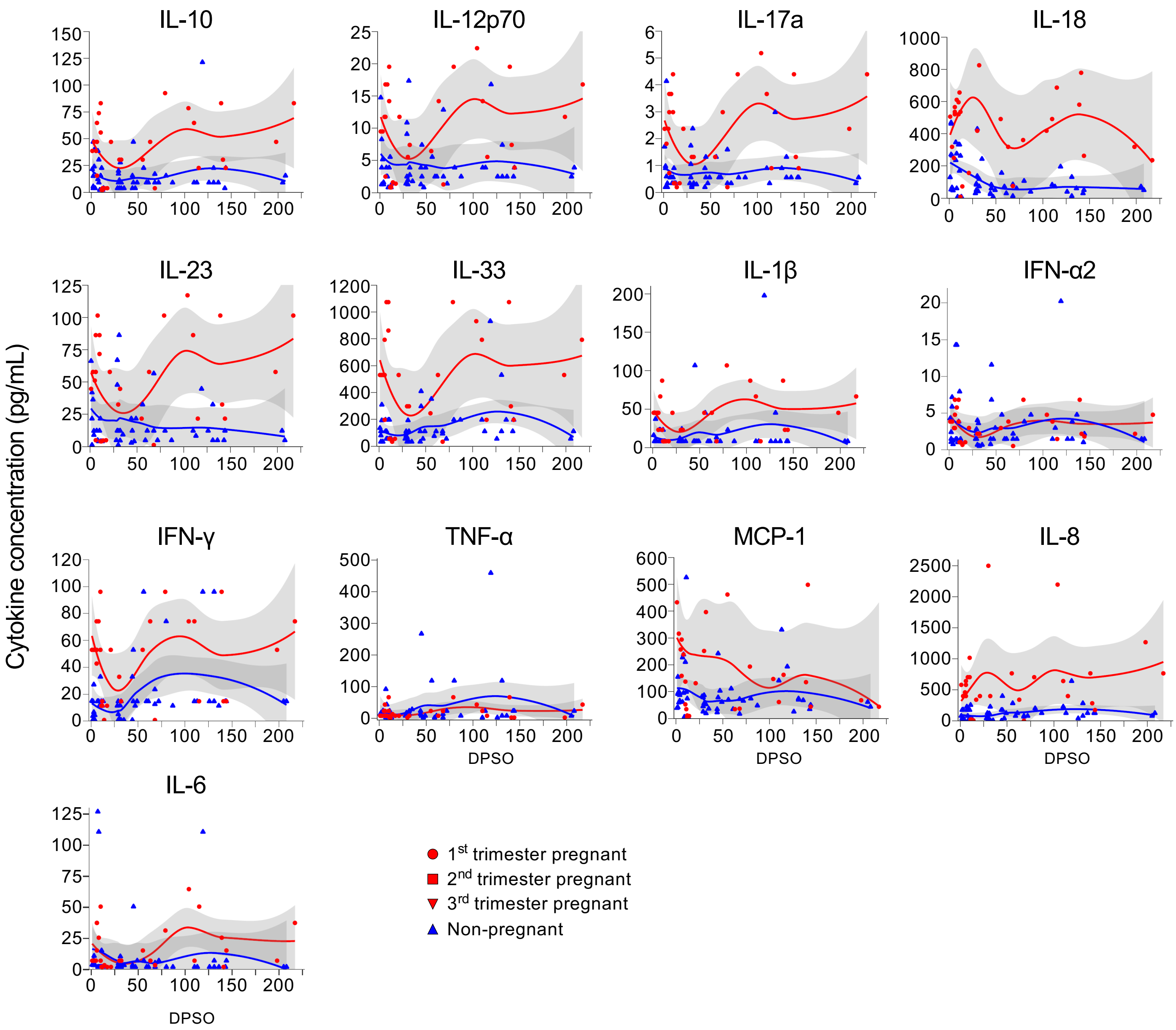
IL-10
IL-12p70
IL-17a
IL-18
IL-23
IL-33
IL-1β
IFN-α2
IFN-γ
TNF-α
MCP-1
IL-8
IL-6
Cytokine concentration (pg/mL)
DPSO
1st trimester pregnant
2nd trimester pregnant
3rd trimester pregnant
Non-pregnant

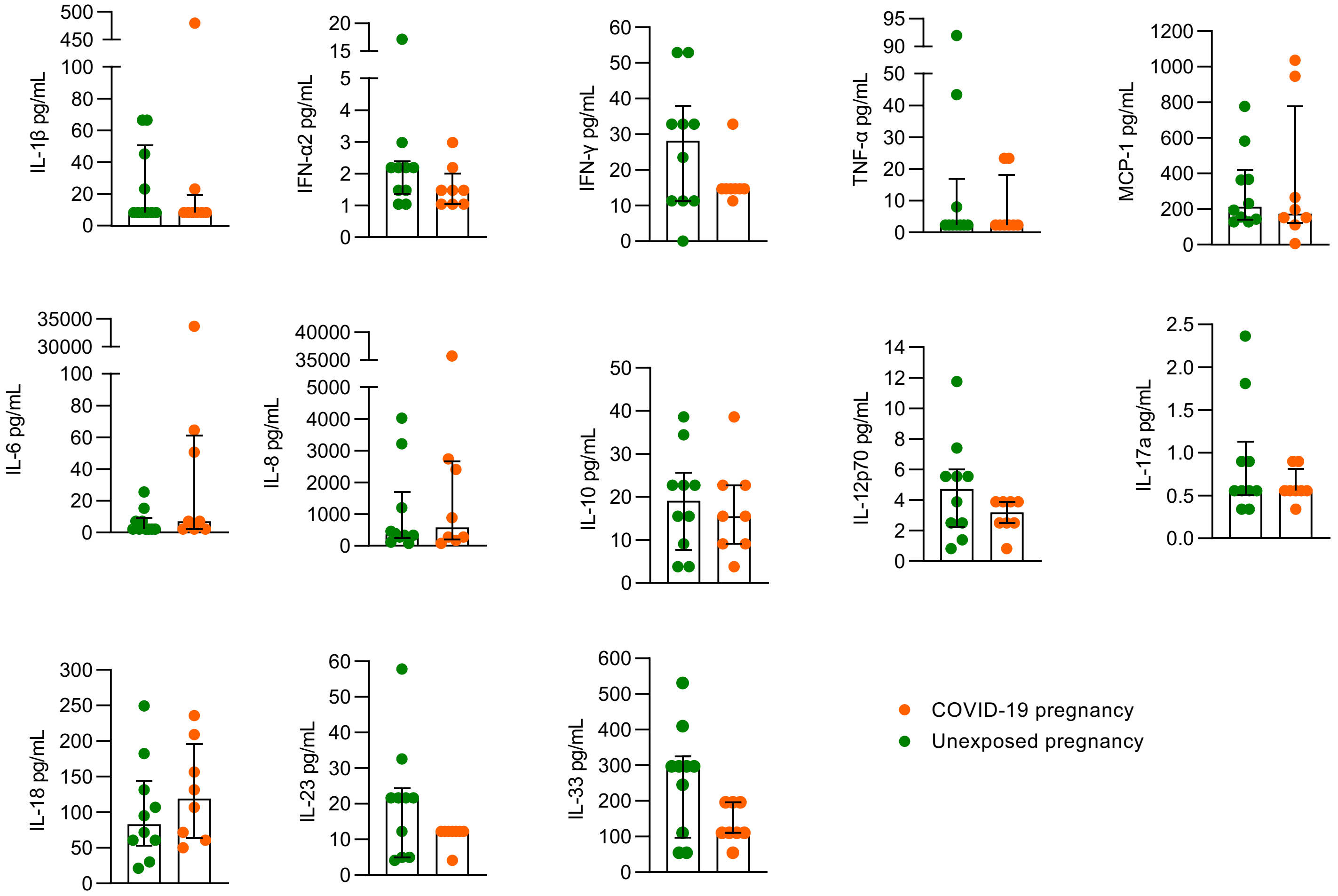

Supplementary Figure 6

A

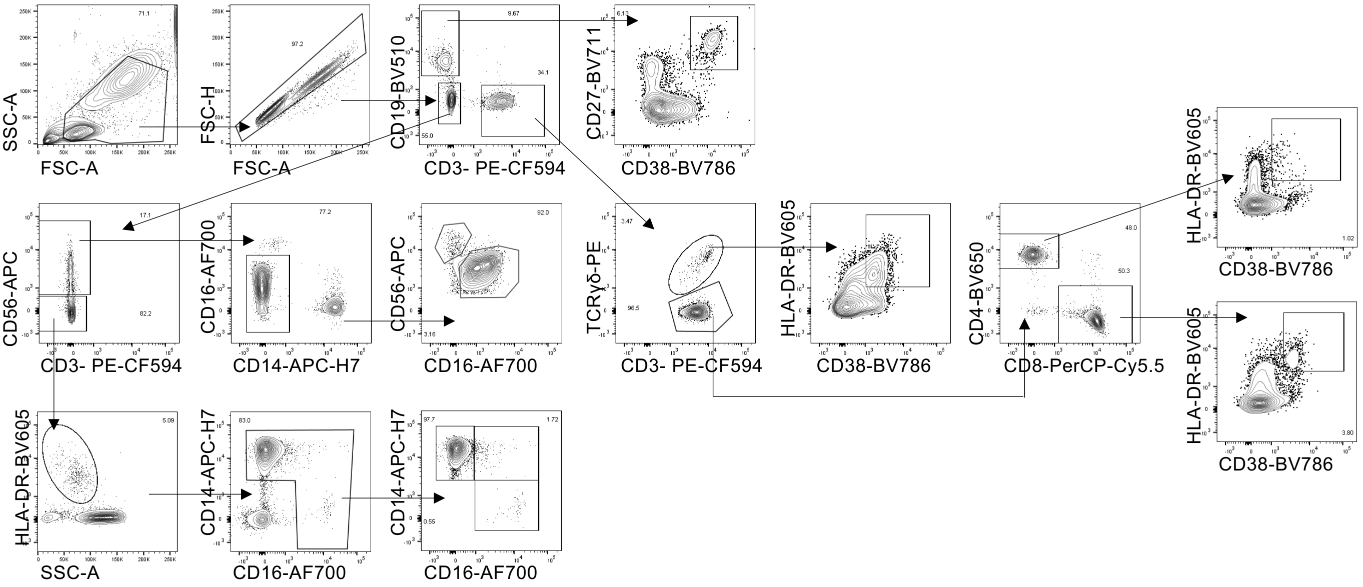

B

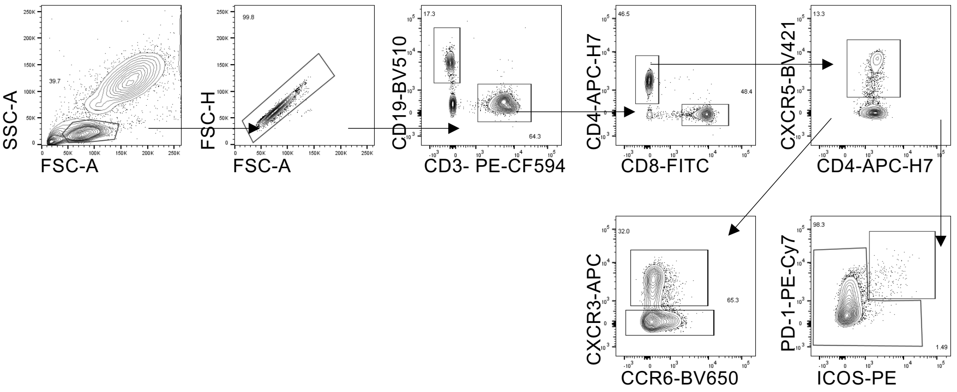

Supplementary Figure 7

Habel et al.

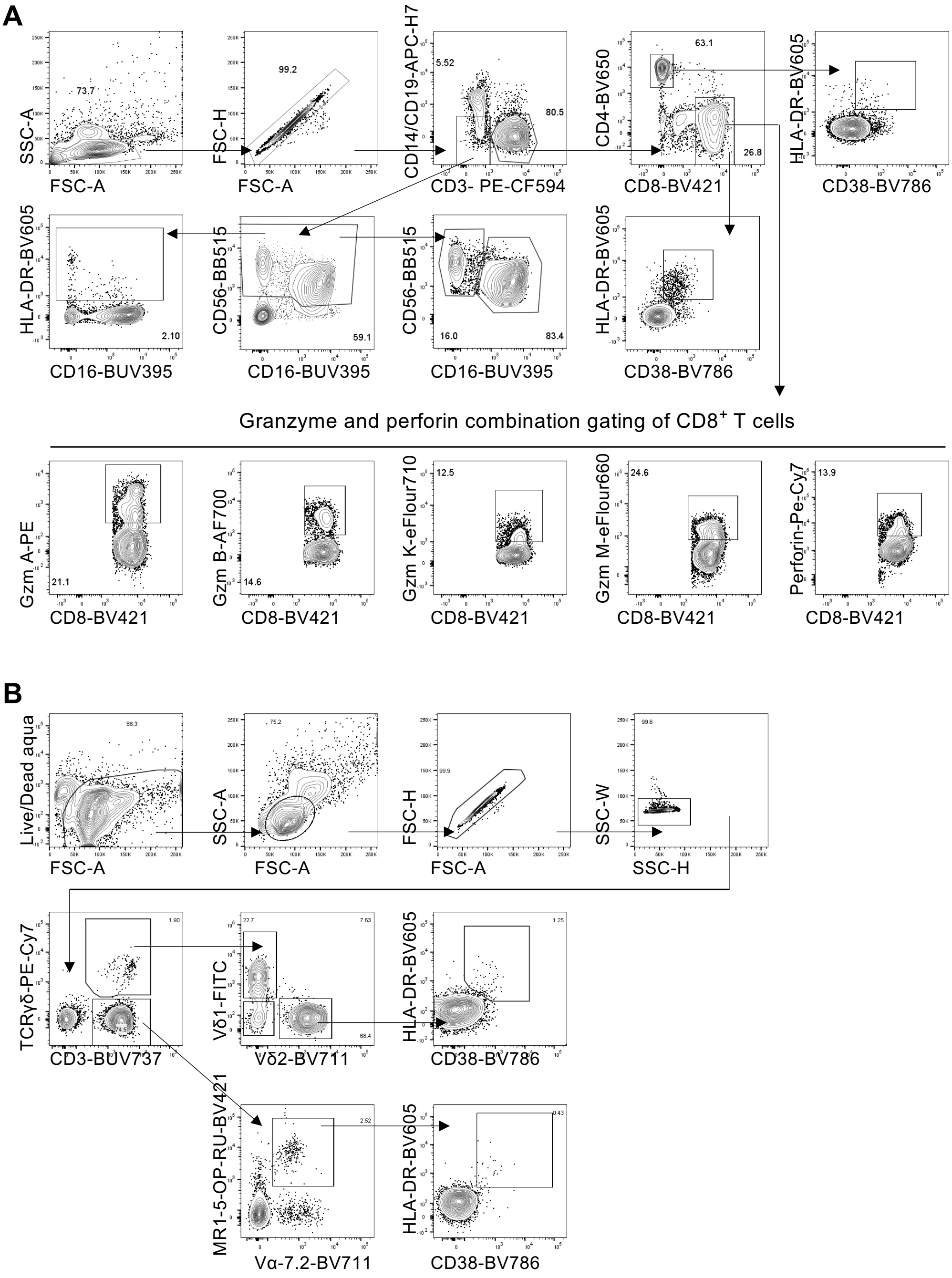

Supplementary Figure 8

Habel et al.
